# Cell Surface Profiling Using High-Throughput Flow Cytometry: A Platform for Biomarker Discovery and Analysis of Cellular Heterogeneity

**DOI:** 10.1371/journal.pone.0105602

**Published:** 2014-08-29

**Authors:** Craig A. Gedye, Ali Hussain, Joshua Paterson, Alannah Smrke, Harleen Saini, Danylo Sirskyj, Keira Pereira, Nazleen Lobo, Jocelyn Stewart, Christopher Go, Jenny Ho, Mauricio Medrano, Elzbieta Hyatt, Julie Yuan, Stevan Lauriault, Maria Kondratyev, Twan van den Beucken, Michael Jewett, Peter Dirks, Cynthia J. Guidos, Jayne Danska, Jean Wang, Bradly Wouters, Benjamin Neel, Robert Rottapel, Laurie E. Ailles

**Affiliations:** 1 Princess Margaret Cancer Centre, University Health Network, Toronto, ON, Canada; 2 Dept. of Medical Biophysics, University of Toronto, Toronto, ON, Canada; 3 Hospital for Sick Children Research Institute, Toronto, ON, Canada; Deutsches Krebsforschungszentrum, Germany

## Abstract

Cell surface proteins have a wide range of biological functions, and are often used as lineage-specific markers. Antibodies that recognize cell surface antigens are widely used as research tools, diagnostic markers, and even therapeutic agents. The ability to obtain broad cell surface protein profiles would thus be of great value in a wide range of fields. There are however currently few available methods for high-throughput analysis of large numbers of cell surface proteins. We describe here a high-throughput flow cytometry (HT-FC) platform for rapid analysis of 363 cell surface antigens. Here we demonstrate that HT-FC provides reproducible results, and use the platform to identify cell surface antigens that are influenced by common cell preparation methods. We show that multiple populations within complex samples such as primary tumors can be simultaneously analyzed by co-staining of cells with lineage-specific antibodies, allowing unprecedented depth of analysis of heterogeneous cell populations. Furthermore, standard informatics methods can be used to visualize, cluster and downsample HT-FC data to reveal novel signatures and biomarkers. We show that the cell surface profile provides sufficient molecular information to classify samples from different cancers and tissue types into biologically relevant clusters using unsupervised hierarchical clustering. Finally, we describe the identification of a candidate lineage marker and its subsequent validation. In summary, HT-FC combines the advantages of a high-throughput screen with a detection method that is sensitive, quantitative, highly reproducible, and allows in-depth analysis of heterogeneous samples. The use of commercially available antibodies means that high quality reagents are immediately available for follow-up studies. HT-FC has a wide range of applications, including biomarker discovery, molecular classification of cancers, or identification of novel lineage specific or stem cell markers.

## Introduction

Cell surface proteins are of particular interest as biomarkers because they perform many important biological functions, including mediation of cell-cell communication and responses to external signals such as the presence of pathogens or chemical messengers. The cell “surfaceome” defines phenotypic and functional differences between cell types, and between normal and diseased cells, such as cancer cells. Cell surface proteins are useful as diagnostic markers or therapeutic targets in cancer, as evidenced by the large number of monoclonal antibodies (MAbs) currently approved for both diagnostic and therapeutic applications. Rapid characterization of the cancer cell surfaceome could not only lead to identification and development of new diagnostic markers and therapeutic targets, but also provide insight into the basic biology of disease, including environmental interactions and identification of important cellular subtypes and signaling pathways.

One approach to cell surfaceome characterization is to bioinformatically predict all membrane proteins in the human genome, and then identify subsets expressed in a given cell type using global gene expression data [1]. However, gene expression does not always correlate with protein expression [2], [3] and not all expressed membrane proteins are present on the cell surface. Another approach has been to perform mass spectrometry-based proteomics, to sensitively and rapidly identify and quantify large numbers of peptides or proteins in a sample of interest. However, this is technically challenging due to the limited abundance of surface membrane proteins, and difficulty obtaining plasma membrane isolates and resolving and identifying hydrophobic proteins and peptides [4]. Recent technical advances have enabled “cell surface-capturing” for more accurate measurement of cell surface proteins by mass spectrometry [4]–[6]. Of note, both whole cell lysate and cell surface capture methods provide an average quantity of molecules measured over the entire sample, making analysis of tissue heterogeneity a challenge.

MAbs can provide reliable information about the expression of cell surface proteins, as well as the distribution of proteins within a heterogeneous tissue. Both immunohistochemistry (IHC) and flow cytometry (FC) utilize chemically- or fluorescently-tagged MAbs to detect proteins, including surface proteins. These assays are specific, sensitive and reproducible, and can provide information at the level of individual cells. However, IHC is limited by the small number of MAbs that can be simultaneously analyzed on a limited number of cells. FC is higher-throughput, allowing rapid analysis of proteins on large numbers cells in liquid suspension. FC is typically used to analyze up to 11 markers at a time, with complex analysis being possible through the use of overlapping panels [7] allowing identification and analysis of subpopulations of cells within complex mixtures. Indeed, such flow cytometry assays are now used clinically in several areas such as diagnosis and monitoring of hematological malignancies [8], [9], illustrating the power of this approach.

Given the importance of cell surface proteins to a wide range of biological processes, their broad utility as research and clinical tools for the identification of specific cell types, their utility as biomarkers of disease, and their potential as therapeutic targets, we wished to develop a cell surface-targeted “array” as a discovery tool. As flow cytometry represents a robust and rapid platform for cell surface protein expression analysis, we assembled the broadest possible panel of fluorochrome-conjugated cell-surface targeted antibodies. These antibodies were arrayed into 96-well plates, allowing high-througphut flow cytometry analysis of their expression using a commercially available high-speed sample loading device for flow cytometers (Becton Dickinson). This high throughput flow cytometry (HT-FC) assay provides highly reproducible results and can be used to answer a wide range of biological questions (see [10] for first published example). We have used the platform to identify cell surface antigens that are influenced by common cell preparation methods such as enzyme digestion, cryopreservation and fixation. By co-staining cells with up to 5 additional MAbs conjugated to complementary fluorochromes, we can segregate populations of cells to allow independent analysis of subpopulations of interest within complex cell mixtures such as primary tumors. Application of standard visualization methods can be used to generate heatmaps and perform supervised or unsupervised hierarchical clustering. We show that the 363-antigen cell surface profile provides sufficient molecular information to classify 119 samples from a variety of cancers and tissue types into biologically relevant clusters using unsupervised hierarchical clustering. Finally, we describe the identification of a candidate lineage-specific marker and its subsequent validation.

## Methods

### Cells

Commercially available cell lines used included MDA-MB-231, SCC4, SCC9, A549, CACO2, OVCAR3, OV-90, 786-O, 22RV1, A2058, H929, 8226, MCF7, and Jurkat, and were obtained from ATCC. Additional cell lines were established in-house in the Ailles Lab (clear cell renal cell carcinoma (ccRCC) cell lines, cancer-associated fibroblasts (CAFs)) or obtained from the labs of colleagues at the University Health Network (serous ovarian cancer (SOC) cell lines, Dr. Rob Rottapel; head and neck cancer (HNC) cell lines, Dr. Bradly Wouters; TEX acute myeloid leukemia cells [11], Dr. Aaron Schimmer). All samples used in this study were obtained from the University Health Network. Written informed consent was obtained for the collection of all human materials, which were used either to generate cell lines or to analyze primary samples directly, and the studies were approved by the Research Ethics Board of the University Health Network. Peripheral blood mononuclear cells (PBMCs) and normal dermal fibroblasts were purchased from Lonza. Cultured cells were quality controlled for cell line identity by short-tandem repeat analysis (AmpFℓSTR Identifiler, Life Technologies), performed by The Centre for Applied Genomics, The Hospital for Sick Children, Toronto, Canada, and tested negative for *Mycoplasma* infection by MycoAlert (Lonza) according to the manufacturer’s instructions. Cell lines were cultured in Iscove’s Modified Dulbecco’s Medium (IMDM) supplemented with 10% fetal bovine serum (FBS; Sigma-Aldrich) and penicillin and streptomycin (Gibco) at final concentrations of 100 U/mL and 100 µg/mL, respectively. Cells were incubated at 37°C in 5% CO_2_, 95% air for commercial cell lines, or 5% CO_2_, 2% O_2_ for primary ccRCC cell lines and CAFs. Adherent cells were passaged using 0.05% trypsin when confluent. Non-adherent cell lines were collected by aspiration and washing in PBS before re-suspension. Primary B-ALL and AML samples were obtained from consenting patients and processed by the Guidos Lab and the Wang Lab, respectively. HNC and SOC tissue samples were obtained from the University Health Network Tissue Bank and from the Cooperative Health Tissue Network. Tumors were procured within 2–24 hours of excision (Cooperative Health Tissue Network samples were shipped overnight from various sites in the United States). Tissues were minced with sterile scalpels and digested with 1X collagenase/hyaluronidase (Stem Cell Technologies) and DNase I (125 U/mL, Invitrogen) in Media199 at 37°C for a maximum of two hours with frequent gentle trituration. In the case of ascites samples from SOC patients, cells were harvested by centrifugation. Red blood cells were lysed by applying 1 mL of ammonium chloride lysis buffer (Gibco) to the cell pellet for 5 minutes on ice, followed by an immediate wash in IMDM/10% FBS. Following red cell lysis, cells were filtered through a 70-micron sterile nylon mesh and viable cells defined by trypan blue exclusion were enumerated at 10X magnification by hemocytometer. To generate CAFs, HNC and SOC single cell suspensions were cultured in IMDM/10% FBS in which CAFs selectively grew and were passaged by trypsinization when confluent. CAF cultures were validated by immunofluorescent staining for vimentin and pan-cytokeratin (CK), as well as by single nucleotide polymorphism array (for HNC CAFs) or p53 immunofluorescence in comparison to matched primary tumors (for SOC CAFs). Samples were either directly used for experiments or cryopreserved in 90% FBS/10% dimethyl sulfoxide.

The pool of cells for testing of enzymatic digestion, cryopreservation and fixation included the following: A549, CACO2, OVCAR3, A2058, SCC4, SCC9, H929, 8226, MCF7, Jurkat, LCL, RCC22, HNC CAFs, and PBMCs. Adherent cells were collected from culture by gentle cell scraping, followed by trituration and 70 µm mesh filtration to form a single cell suspension. Cells were mixed in equivalent doses (2 million each) to generate a mixed cell lineage. Aliquots of this cell mixture were then stained with no manipulation (control), stained and then fixed, subjected to enzymatic treatment then stained, or cryopreserved. Enzymatic digestion for the initial screen was performed by resuspending the cell aliquot in IMDM without serum and adding a mixture of collagenase type IV (Worthington Biochemical, 48J106-42, final concentration 200 U/mL), hyaluronidase type IV-S (Sigma Aldrich, H3884, final concentration 100 U/mL) and deoxyribonuclease I (Worthington Biochemical, 57S10106002, final concentration 100 U/mL). Cells were incubated at 37°C for 20 minutes whereupon dispase, (Becton Dickinson, 354235, final concentration 2.5 U/mL) was added followed 5 minutes later by 0.25% trypsin (Gibco, 25200-056, final concentration 0.05%) for a final 5 minutes. The cell suspension was then washed in IMDM/10% FBS to inactivate trypsin, followed by a further two washes in PBS. Digested cells were then resuspended in Hank’s balanced salt solution+1% bovine serum albumin, 2 mM EDTA (FC buffer) and kept at 4°C during antibody staining. Cells were cryopreserved by resuspending in FBS containing 10% dimethyl sulfoxide and freezing in a CoolCell container (Biocision) to –80°C overnight before transferring to vapour phase liquid nitrogen storage at –140°C. Vials were stored for 4 weeks, then rapidly thawed and slowly diluted with PBS, washed twice with PBS and then resuspended in FC buffer and stained. Post-staining fixation with paraformaldehyde was performed by centrifuging the 96-well plates containing the stained cells (see below) and resuspending cell pellets in BD Cytofix (Becton Dickinson) diluted 1∶3 with PBS. Cells were incubated on ice for 30 minutes, then washed with PBS and resuspended in FC buffer. Validation of antigens that appeared to be affected by a preparatory step was performed by generating a custom plate of the relevant antibodies, performing the preparatory steps, and staining as above. Antigens that appeared to be affected by digestion were tested against each enzyme individually.

### HT-FC staining protocol

A schematic diagram of the staining protocol is shown in Figure S1. 363 commercially available directly fluorochrome-conjugated antibodies to cell surface antigens conjugated to PE, FITC or APC (see Table S1) were purchased and aliquoted into round bottom 96-well plates (2 µl per well into 48 µl of FC buffer). Control wells with FC buffer alone were included. Cell suspensions of 0.5 to 1 million cells/mL were aliquoted by multichannel pipette into pre-prepared HT-FC plates (50 µl per well), for a final volume of 100 µl per well and a final antibody dilution of 1∶50. Plates were incubated for 20 minutes on ice in the dark, centrifuged for 5 minutes at 350×g, washed twice with 200 µl FC buffer, and resuspended in 50 to 80 µl FC buffer containing 0.1 µg/mL 4′,6-diamidino-2-phenylindole (DAPI; Sigma-Aldrich). In experiments where cells were fixed after antibody staining, viability was assessed with the LIVE/DEAD fixable near-IR cell stain according to the manufacturer’s instructions (Invitrogen). In parallel, aliquots were stained in tubes for fluorescence-minus-one controls, which consisted of DAPI only staining if no co-staining was done. Antibodies used for co-staining of primary tumor samples included CD45-APC-Cy7 (1∶200), CD31-PE-Cy7 (1∶200), CD34-PerCP-Cy5.5 (1∶50) (all BioLegend) and 1.2 µg/ml TE7-biotin (in-house production from hybridoma obtained from ATCC) followed by Streptavidin-eFluor450 (1∶400; eBioscience). Fluorescence-minus-one controls were generated for each antibody used and compensations were set using BD Plus CompBeads and FACSDiva software. Data collection was performed on a Becton-Dickinson LSR II flow cytometer with ultraviolet (20 mW), violet (25 mW), blue (20 mW) and red (17 mW) lasers, with default filter configuration, utilizing the High Throughput Sampler attachment. At least 10,000 events were collected per well. The gating strategy based on fluorescence-minus-one controls is illustrated in Figures S2 and S4.

### Data analysis

FCS 3.0 files were exported to FlowJo version 9.3. In all cases, dead cells and doublets were excluded prior to analyzing marker expression. Gates were set using fluorescence-minus-one controls, and percent-positive values were exported to Excel for use in further statistical analysis, generation of heat maps and hierarchical clustering. Spearman correlation coefficients and p values were calculated using GraphPad Prism software. Heat maps, hierarchical clustering and principle components analyses were performed using MultiExperiment Viewer software (www.tm4.org). Unsupervised hierarchical clustering was performed using a Pearson correlation distance metric with complete linkage clustering. Principle components analysis used a mean centering mode and 10 neighbors for *k*-nearest neighbors imputation.

### Immunofluorescence

Frozen sections of SOC samples were co-stained for pan-CK and CD90, as follows: sections were fixed in ice-cold acetone, air-dried and blocked with 0.5% bovine serum albumin and 5% goat serum. Purified pan-CK antibody (1∶200; Abcam clone C-11) was applied and incubated for 1 hour at room temperature. Goat-anti-mouse-Alexa488 (1∶400; Life Technologies) was applied and slides were incubated in the dark for 30 minutes at room temperature. The slides were then washed in PBS/0.1% Tween-20 prior to incubation with CD90-biotinylated antibody (BD Pharmingen, 1∶200) for one hour at room temperature. Finally, Streptavidin-Alexa594 (Life Technologies) was applied at 1∶400 in the dark. The sections were then stained with Hoechst for one minute prior to imaging using a Leica DMI6000 fluorescent microscope. p53 staining on purified SOC populations was carried out by first staining cells in suspension with 1∶200 each of CD45/CD31-PE-Cy7 as described above, plus 1∶100 EpCAM-APC (BioLegend) and 1∶200 CD90-PE (BD Pharmingen). The CD45^−^CD31^−^ population was fractionated into the EpCAM^+^ population and the EpCAM^−^CD90^++^ population and cells were purified using fluorescence activated cell sorting on an Becton Dickinson FACS Aria II with blue (15 mW), red (15 mW) and violet (10 mW) lasers, with default filter configuration. Purified cells were cytospun onto glass slides, fixed in ice-cold methanol and stained for p53 as above, using clone DO-7 (1∶50, Santa Cruz Biotech).

## Results

### Platform Reproducibility

**Figure 1:**
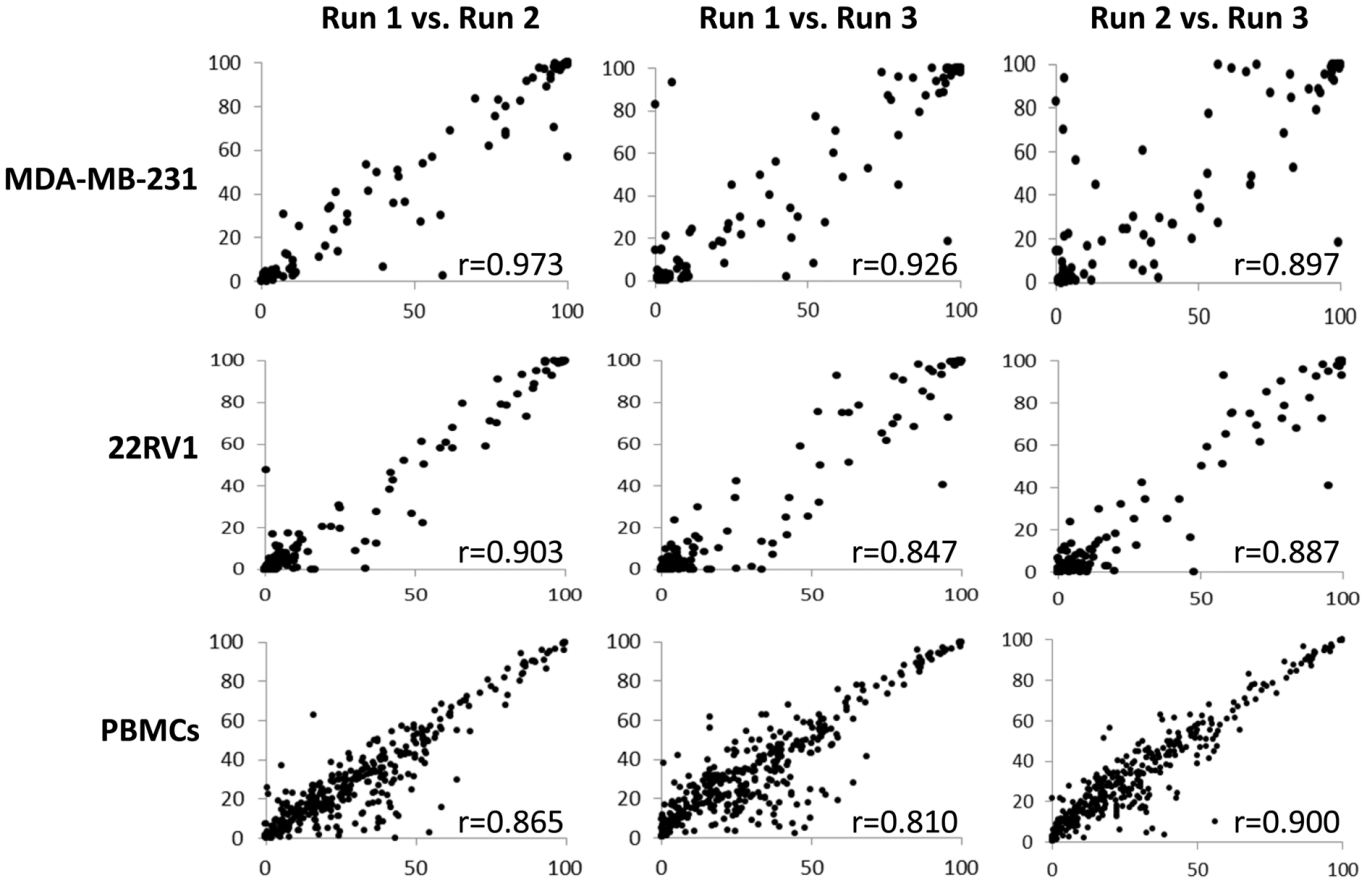
The HT-FC platform is highly reproducible. MDA-MB-231 breast cancer cells, 22RV1 prostate cancer cells, or PBMCs were each run 3 times on the HT-FC platform. When the same cell line or peripheral blood sample was analyzed 3 independent times, consistently reproducible results were obtained, with Spearman correlation coefficients between runs ranging from 0.847 to 0.973, and p<0.001 for all comparisons.

To facilitate screening of large numbers of antibodies rapidly, we took advantage of the recent development of a high-speed sample loading device for flow cytometers that allows automated acquisition of samples from multi-well plates. We identified and purchased 363 antibodies (Table S1), which represented all that were commercially available at that time. These were aliquoted into 96-well plates to generate what is in essence an antibody array. We initially performed single-color staining on peripheral blood mononuclear cells and cell lines by simply adding cells into the wells and performing all antibody incubation and washing steps in the plates (see Supplementary Figure S1 for a schematic of the work flow). Control wells containing no antibody were included. Cell staining and data acquisition could be performed in a period of 4 to 5 hours total. Non-viable cells were detected by DAPI staining and cell doublets and clusters were eliminated by forward scatter and side scatter height *vs.* width plots. A gate was then set on the control “fluorescence-minus-one” sample (see Methods and Figures S1 and S2) and a table of percent-positive values for all the antibodies was generated. To test the reproducibility of the HT-FC platform, we performed triplicate runs on a number of different sample types. Figure 1 shows the correlation of percent-positive values for multiple runs performed on MDA-MB-231 breast cancer cells, 22RV1 prostate cancer cells, and PBMCs. In all cases, the reproducibility of marker detection was very high, with Spearman correlation coefficients (r^2^) ranging from 0.81 to 0.97 (p<0.001 for all comparisons).

### Identification of antigens that are altered by common sample preparation methods

Flow cytometry is a technique that is commonly used to analyze cell surface antigens on cells that have been subjected to various experimental manipulations in the lab. These include enzyme digestion, such as trypsinization of cultured cells or collagenase digestion of solid tissues, and cryopreservation. In addition, it is common to fix cells in paraformaldehyde prior to analysis, in order to preserve cells until convenient to analyze and/or eliminate potential biohazards present in human samples. Cell surface proteins are potentially vulnerable to such treatments and may be negatively affected by non-specific enzymatic digestion and/or altered in response to stressful conditions such as freezing and thawing. We therefore sought to determine the influence of these common manipulations on cell surface antigen detection.

**Figure 2:**
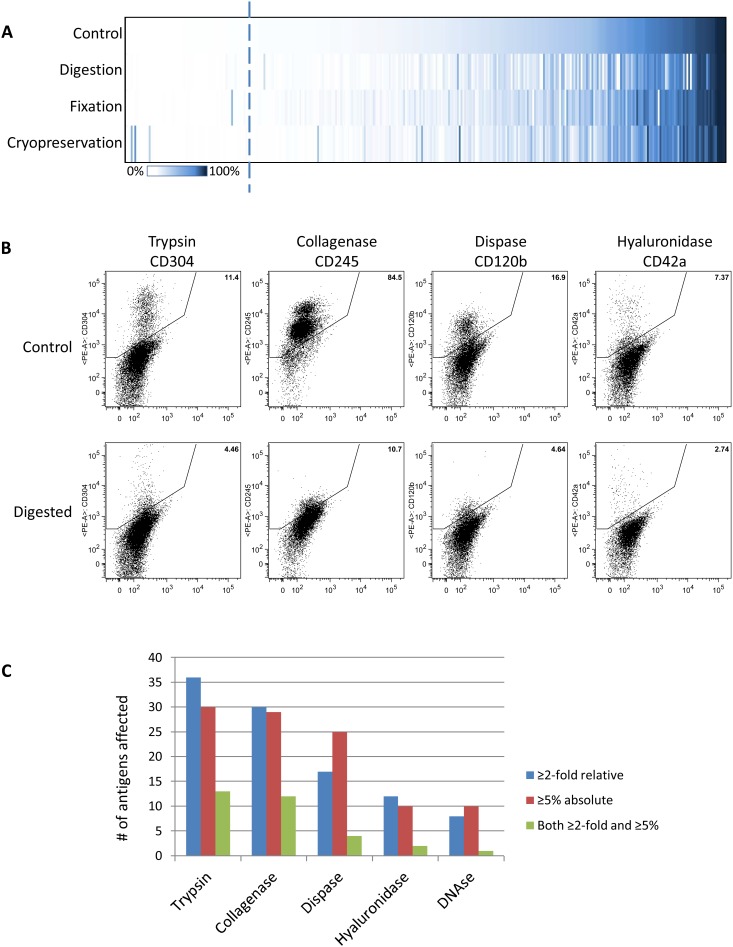
Effect of typical experimental procedures on HT-FC antigen detection. (**A**) 267 out of 344 antigens showed expression of greater than 1% on the “untreated” cell pool (nominal limit of detection of 1%; dashed line). Digestion with a cocktail of enzymes containing trypsin, collagenase, hyaluronidase, dispase and DNAse affected the largest number of antigens, while fixation and cryopreservation affected fewer antigens. (**B**) Examples of validated antigen loss after digestion with individual enzymes trypsin (CD304), collagenase (CD245), dispase (CD120b) and hyaluronidase (CD42a). (**C**) Collagenase and trypsin were typically the most influential enzymes, but all had some influence. Graph shows the number of antigens altered by ≥2-fold relative to untreated cells (blue), or by ≥5 percent-positive cells (red), or both 2-fold and ≥5 percent-positive cells (green). Data for all enzymes is provided in Table S2.

A variety of cell types were sourced and pooled in an attempt to generate the largest possible coverage of epitope detection. These included PBMCs, epithelial cancer cell lines, melanoma cell lines, leukemia cell lines, and cultured fibroblasts. All cell lines were confirmed to be *Mycoplasma* negative and identity was verified by short tandem repeat profiling [12]. This pool of cells was harvested without enzymes (adherent cells were detached using EDTA and gentle scraping and trituration), treated with a cocktail of trypsin, collagenase, hyaluronidase, dispase and DNAse with appropriate incubation times, or cryopreserved. Experiments were also done to compare unfixed cells to cells that were fixed in 2% paraformaldehyde in PBS after staining. At the time of these experiments, the panel contained 334 antibodies. Overall 258 of 334 antigens were expressed on ≥1% of the pooled cells (Figure 2A), therefore we could not determine the sensitivity of 76 antigens to the various preparation methods. Of the 258 expressed, a number of antigens showed altered expression after exposure to enzymatic digestion, fixation or cryopreservation, as seen in the heat map in Figure 2A (based on percent-positive values). Enzymatic digestion with a pooled enzyme cocktail influenced 79 antigens (30% of 258 antigens detectable) in our preliminary screen, and these were subsequently taken forward for validation against individual enzymes in a customized plate. Of these 79 antigens, 39 were validated as showing an absolute change in percent-positive cells of at least 5% with at least one enzyme, suggesting that many altered in the original screen were falsely influenced, likely due to combination treatment within the enzyme cocktail. In total, 22 antigens showed both an absolute detection change of at least 5% and a fold-change of at least 2-fold with at least one enzyme (Tables S2 and S3). Some specific examples of observed changes are shown in Figure 2B. Interestingly, many of these have known extracellular cleavage sites and are biologically active upon release from the cell surface (Table S3). Of the individual enzymes, trypsin and collagenase demonstrated the greatest influence, with dispase and hyaluronidase showing less influence (Figure 2C). However no enzyme was spared and even DNase alone appeared to show some modest alterations in detection of some antigens. Individual enzymes had different effects on different antigens (Table S2).

Fixation with paraformaldehyde after antibody staining, and cryopreservation showed the least overall perturbations of antigen detection in the preliminary screen; 31 and 40 antigens, respectively, were altered by either ≥5% positive cells or ≥2-fold. Upon validation, only 8 antigens showed significant variation of more than 5% absolute and 2-fold relative difference when fixed (Table S4). Similarly, only 9 antigens showed a significant change when cryopreserved (Table S5). Interestingly, only one antigen showed significant loss of expression upon cryopreservation, while the other 8 were increased.

### Cell surface profiles stratify samples into biologically relevant clusters

We have run the HT-FC platform on a wide range of cell types, including many epithelial cancers (traditional and novel cell lines, primary samples), normal and malignant hematopoietic cells, fibroblasts from various sources, and a small selection of normal and malignant primary neural stem cell cultures. We identified only 10 antibodies that never stained any cell type analyzed; of these, 5 are predicted to stain very specific cell types that were not among those tested by us, and 5 should have stained cells that we tested and did not, suggesting that these 5 antibodies are suboptimal for this application (Table 1). Of the remaining antibodies, in addition to staining the predicted cell types, there were many that stained cell types not previously identified as expressing those markers. For example, some markers stained virtually every cell type we examined, while other antibodies were very cell-type or lineage specific.

**Table 1:** Antibodies with no staining across all cell types analyzed.

Antigen	Expected Cell Type	Cell Type Analyzed?
CD103	intestinal intraepitheliallymphocytes (IEL)	No
CD41b	platelets	No
PAC-1	platelets	No
CD1a	cortical thymocytes, dendriticcells and Langerhans cells;DOES NOT react with PBMCs	No
CD209	dendritic cells, vascular endothelial cells	No
CD150	T cells, B cells, thymocytes,germinal center and dendritic cells	**Yes**
CD16b	neutrophils	**Yes**
FMC7	B lymphocytes	**Yes**
CD197	lymphocytes	**Yes**
CD129	low levels on eosinophils,mast cells, macrophages, Blymphocytes, T lymphocytes,and erythroid progenitors	**Yes**

**Figure 3:**
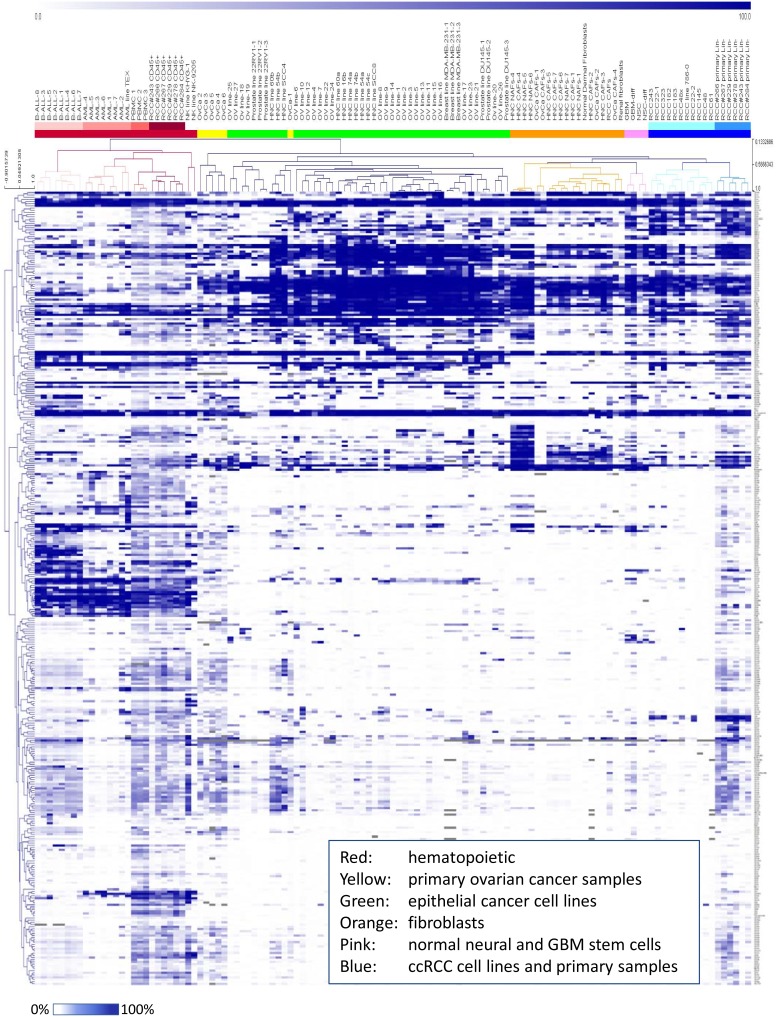
HT-FC allows phenotypic segregation and identification of cell samples from diverse lineages. Unsupervised hierarchical clustering of percent-positive marker expression values generated on 119 samples was performed. Colours indicate emergence of biologically related samples into clusters based on surface marker profiles. See Figure S2 for a magnified image of the dendrogram.

Gene expression profiling has been widely used to generate “molecular signatures” that define a particular cell or tissue type or that can be used to define molecular subtypes within a given disease that are associated with clinical behaviors or outcomes [13]–[16]. To determine whether our 363-protein profile could similarly stratify samples based on their cell of origin, we compiled the large amount of HT-FC data obtained across many cell types using percent-positive values to perform unsupervised hierarchical clustering (Figure 3 and Figure S3). We found that the cell surface phenotype stratifies samples into clusters of related cell types or lineages. For example, we see the following major clusters: a hematopoietic cluster (red), which contains sub-clusters that include PBMCs, AML samples, B-ALL samples, and the CD45+ immune cell fraction of primary ccRCC samples; an epithelial cancer cell line cluster (green) composed of multiple sub-clusters; a fibroblast cluster (orange) which includes CAFs derived from SOC and HNC and normal dermal fibroblasts; and a ccRCC cluster (blue), which contains both early passage in-house cell lines and primary samples. Primary SOC samples form an independent cluster (yellow), whereas ccRCC cell lines and primary samples cluster together and are more closely associated with neuronal cells (pink) and fibroblasts (orange) than with epithelial cell lines. Principal components analysis revealed similar clustering patterns (Figure S3). These results indicate that the 363 cell surface protein profile provides sufficient molecular information to segregate human samples into biologically relevant groups, and could be used to rapidly identify cell surface proteins associated with specific cell types or disease states. Table S6 contains data obtained for PBMCs, MBA-MB231 cells, 22RV1 cells, renal cancer samples and cell lines, and CAFs derived from ovarian cancer and head and neck cancer primary samples.

### Interrogation of cellular heterogeneity in primary tissue samples

**Figure 4:**
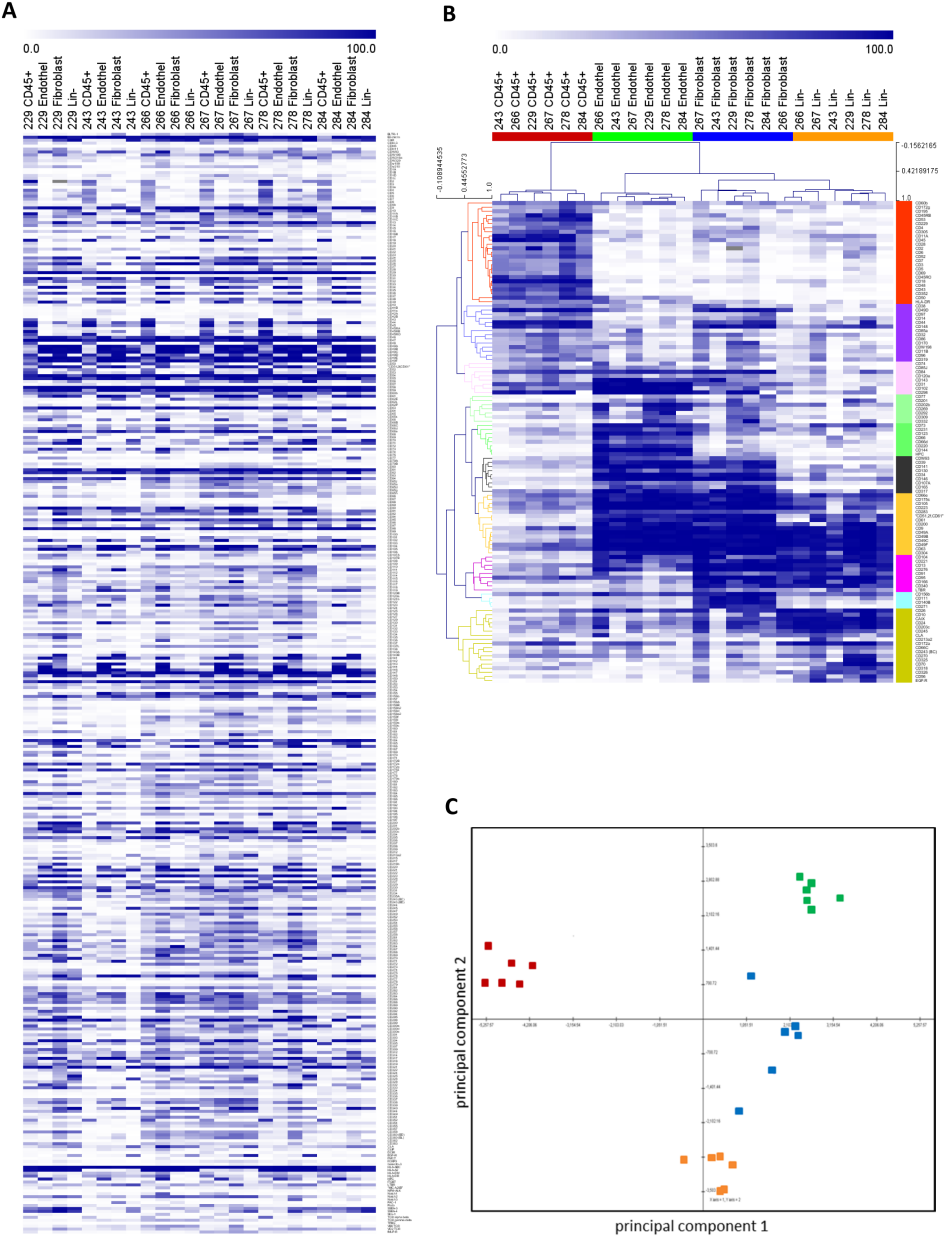
HT-FC allows intratumoral analysis of stromal and cancer cell subsets within primary ccRCC samples. (**A**) Heatmap showing expression of each of the 363 antibodies in four subpopulations of ccRCC samples: CD45^+^ immune cells, CD45^−^CD31^+^CD34^+^ vascular endothelial cells, CD45^−^TE7^+^ fibroblasts, and CD45^−^TE7^−^CD31^−^CD34^−^ cancer cells. Antibodies are simply arranged in alphabetical order on the vertical axis and the four populations for each sample are ordered across the top. A low resolution overview demonstrates a surprisingly reproducible “fingerprint” of tumor cell subpopulations from one sample to the next. (**B**) Supervised hierarchical clustering reveals clusters of antigens corresponding to specific cell subsets within tumors (see Table S6 for details). (**C**) Principal components analysis of the entire data set further illustrates how effectively the cell surface profile delineates the 4 distinct cell populations within primary ccRCC samples. Red: immune cells; Green: endothelial cells; Blue: fibroblasts; Orange: cancer cells.

Performing HT-FC on cell lines is straightforward, requiring simply aliquoting cells into 96-well plates, where each well contains a single antibody. However, primary tissues that are dissociated into single cell suspensions for FC analysis contain a complex mixture of cell types, and thus can’t be accurately analyzed using a single marker at a time. For analysis of the primary SOC and ccRCC samples shown in Figure 3, samples were co-stained with additional markers to allow identification of abundant immune cells and other stromal cell types that are present in primary tumor samples, and selection of only the cancer cell population for analysis of the cell surface profile. However, co-staining of primary samples with multiple antibodies conjugated to additional fluorochromes not only allowed unwanted stromal cells to be excluded from the analysis of cancer cells within a tumor tissue, but also allowed us to obtain the cell surface profile of multiple populations within these complex samples. For example, the 6 primary patient-derived ccRCC samples were initially stained with four stromal-targeted antibodies prior to staining them with the full antibody panel (Figure 4). From this we were able to generate heat maps representing the cell surface profile of the hematopoietic (CD45^+^), vascular endothelial (CD31^+^/CD34^+^), fibroblast (TE7^+^) [17] and cancer (CD45^−^/CD31^−^/CD34^−^/TE7^−^) cell populations within each tumor. The gating strategy is provided in Figure S4. Figure 4A shows an expression heat map of the data (without hierarchical clustering). Antibodies are arranged in simple alphabetical order on the vertical axis for the four populations within each sample, grouped across the top. It is clear from the heat map that a remarkably similar pattern of staining is obtained from one sample to next, once again illustrating the reproducibility of the platform even when applied to individual patient-derived samples. Upon supervised hierarchical clustering, several antigen clusters were revealed that delineate the different cell lineages analyzed (Figure 4B). Some of the clusters contained predicted markers, such as CD3, CD4, CD9, and CD11b on the immune cell cluster (red on vertical axis); CD141, CD144 and CD146 on endothelial cells (green and black); and CAIX and CD10 on cancer cells (yellow). Interestingly, we also identified markers that have previously not been defined to be specific for the indicated lineages, or that stained multiple cell types (for example, a cluster specific for endothelial cells and fibroblasts, another for immune cells and endothelial cells, *etc.*). The markers for the identified clusters are listed in Table S7.

### Identification of a cancer-associated fibroblast (CAF) cell surface marker in serous ovarian cancer

**Figure 5:**
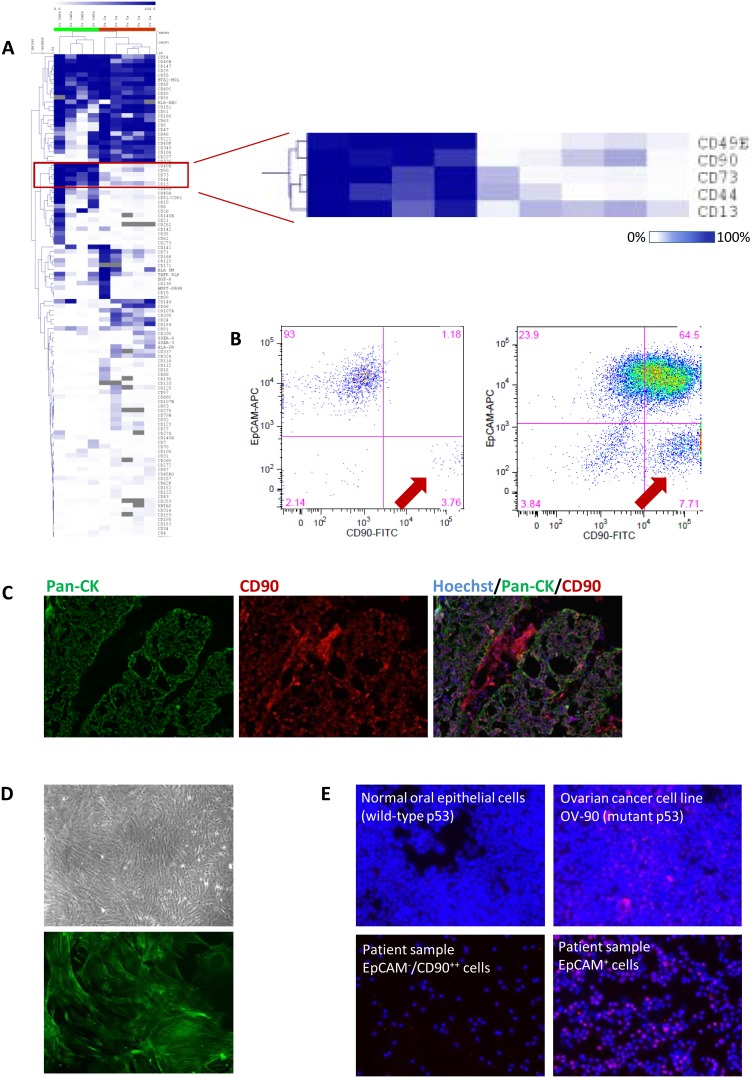
HT-FC identifies CD90 as a CAF marker in serous ovarian cancer. (**A**) HT- FC was performed on 4 SOC-derived CAF lines and 5 primary SOC samples. CD45^+^CD31^+^ cells were excluded from the analysis of the primary samples. A cluster of antigens was identified that were highly expressed in CAFs and expressed at low levels in primary cancer cells. (**B**) FACS plots of two primary SOC samples, gated on viable CD45^−^CD31^−^ cells, showing CD90 *vs*. EpCAM staining. A distinct population of EpCAM^−^CD90^++^ cells is consistently seen, whereas CD90 expression is variable on the EpCAM^+^ cancer cells. Two representative samples are shown, out of a total of 19 samples analyzed. (**C**) Immunofluorescence staining of a frozen section of SOC (same sample shown in FACS plot on right in (B) above), stained with a pan-CK antibody (green) and CD90 (red). CD90 stains both the CK^+^ cells and the CK^−^ stromal cells, with more intense staining on the latter, thus recapitulating the staining seen by flow cytometry. Magnification = 100X. (**D**) FACS-purified EpCAM^−^CD90^++^ cells efficiently establish cultures that display a fibroblast phenotype (top; 40X) and stain positive for CAF marker α-smooth muscle actin (bottom; 100X). (**E**) Immunofluorescence staining for p53 is positive EpCAM^+^ cancer cells, indicative of mutant p53 in this sample (bottom right), whereas the EpCAM^−^CD90^++^ putative fibroblast population from the same sample is p53 negative, and thus wild-type (bottom left). Controls: normal oral epithelial cells (p53 wildtype; top left), p53 mutant ovarian cancer cell line (OV-90; top right). Magnification = 100X.

As with any high-throughput screen it is important to demonstrate its ability to identify specific candidates of interest, which are then validated upon follow-up investigations. Our laboratory has an interest in the role of CAFs in high-grade SOC. While it is possible to generate cultured CAFs from primary SOC samples that then can be passaged *in vitro*, we were interested in identifying cell surface markers for SOC stromal fibroblasts to facilitate their purification directly from primary tumors, allowing us to avoid artifacts associated with culturing cells for multiple passages. While many CAF markers are available (*e.g.* Vimentin, α-smooth muscle actin), the majority of them are intracellular, and thus cannot be used to purify viable cells using fluorescence-activated cell sorting (FACS). Fibroblast activation protein and platelet-derived growth factor receptor-β are reported to be cell surface fibroblast markers, but despite employing multiple different antibodies at high dilution we found the expression of both of these markers to be low and/or not to yield distinct cell populations that would allow for cell sorting of SOC samples. TE7, the fibroblast marker used to identify fibroblasts in ccRCC samples described above, was also found to be suboptimal for staining of fibroblasts in SOC. To identify new cell surface fibroblast markers, we performed the HT-FC screen on four cultured CAF lines we had generated, as well as on five primary SOC samples, co-stained with CD45 and CD31 to allow exclusion of contaminating immune and endothelial cell types. We then selected markers that were highly expressed on the pure fibroblasts but expressed at low levels on the CD45/CD31-negative fraction of primary SOC samples, which would be expected to contain predominantly cancer cells and contaminating fibroblasts. Based on this analysis, we identified 5 candidate CAF markers (Figure 5A). We chose to follow up on CD90, as it has been reported by others to be expressed on mesenchymal stem cells [18], on cells resembling mesenchymal stem cells that were cultured from SOC samples [19], and also to be a CAF marker in prostate cancer [20], [21].

To validate CD90 as a candidate SOC CAF marker, we co-stained primary SOC samples with hematopoietic and vascular cell markers (CD45 and CD31), epithelial cell marker EpCAM (epithelial cell adhesion molecule), and CD90, and consistently identified a population of cells that was CD45^−^CD31^−^EpCAM^−^ and very bright for CD90 (CD90^++^; Figure 5B and Figure S5), which we hypothesized to represent the stromal fibroblasts. The proportion of CD45^−^CD31^−^EpCAM^−^CD90^++^ cells varied from sample to sample, ranging from <1% to 56% (mean 10.0%, n = 24). CD90 was also found to be variably expressed on the EpCAM^+^ cancer cells, and thus must be used in combination with EpCAM to cleanly distinguish the stromal fibroblast population. Interestingly, the CD90 expression level was consistently higher on the EpCAM-negative fraction, as indicated by higher mean-fluorescence intensity of this population (Figure 5B). We confirmed the flow cytometry results using immunofluorescence co-staining with a pan-CK antibody (Figure 5C), where we saw brighter staining of stromal cells *vs*. epithelial cells. We also found that cell cultures initiated with FACS-purified CD45^−^CD31^−^EpCAM^−^CD90^++^ cells gave rise to fibroblasts that were positive for α-smooth muscle actin, a classical CAF marker (Figure 5D). Finally, we verified that the EpCAM^−^CD90^++^ cells were not cancer cells by preparing cytospins of the purified cell populations and staining them for p53. The majority of high-grade SOC samples are p53 mutant [22], which is indicated by high positive nuclear staining with a p53 antibody [23]. We therefore performed immunofluorescence staining for p53 on the EpCAM^+^ and EpCAM^−^CD90^++^ fractions from 4 samples. Three of these samples were positive for p53 staining in the EpCAM^+^ fraction, indicating their mutant status; the fourth sample was negative for p53 staining and thus was uninformative. In all three of the p53-mutant tumors, the matching EpCAM^−^CD90^++^ cells were negative and therefore p53 wild-type, verifying that these were not cancer-derived cells that had lost EpCAM expression and/or gained a mesenchymal phenotype *via* epithelial-to-mesenchymal transition. Thus the combination of EpCAM/CD90 staining together with CD45 and CD31 allows for isolation of stromal fibroblasts directly from primary patient samples for further characterization.

## Discussion

Flow cytometry has long been a robust tool for reliable detection of cell surface proteins, and has many advantages over other immunological protein detection methods, such as Western blotting and IHC. These include ease of use, the ability to rapidly analyze very large cell numbers, analysis of rare populations of cells, and the ability to obtain multi-parameter information on individual cells, which is particularly important for heterogeneous cell samples. The robustness and utility of flow cytometry is illustrated by the large number of clinical applications for which it is now being used around the world [9]. Traditionally, flow cytometry assays are performed on individual samples with a panel composed of up to 11 antibodies at a time that are known to be useful for a particular diagnosis or identification of specific cell types. More recently mass cytometry or “cytometry time-of-flight” (CyTOF) assays, in which antibodies are labelled with lanthanide metals rather than fluorochromes and then detected by mass spectrometry, has been developed [24]. Due to the lack of spectral overlap between antibodies, this allows an even greater degree of multiplexing to be performed, currently allowing for simultaneous analysis of up to 35 markers at a time, but with the potential to increase this up to 100 in the future [24], [25]. The HT-FC platform described here is complementary to these methods, as it takes a more unbiased, discovery-oriented screening approach; by screening cells of interest for a large panel of cell surface markers, it is possible to identify previously unknown proteins or protein combinations expressed on the surface of cells of interest, which once identified can then be developed into the more traditional multiplexed flow cytometry assays or translated into CyTOF assays. Thus HT-FC represents a valuable tool for new marker discovery, comparable to other discovery platforms such as gene expression microarrays, but with a focus on cell surface proteins. The cost per sample is similar to or less than gene expression microarrays (depending on the microarray platform), and is significantly less than similar commercially available cell surface protein screens. Each assay can be performed in an afternoon, and there is the potential to multiplex samples by pre-labelling with fluorescent dyes [26] to increase throughput and reduce costs even further. Data analysis is straightforward, as gates that are set on control samples are simply applied to the entire data set and a spreadsheet of expression values is generated that can then be used in standard bioinformatics software packages (we used MultiExperiment Viewer). This is in contrast to CyTOF data, which requires antibodies to be conjugated to lanthanides and optimized by the individual investigator, and for which a simple and straightforward data analysis method is yet to be devised [27], [28].

The use of well-established and validated commercially available antibodies means high quality protein-level data is obtained that will have a very high validation rate; indeed flow cytometry is a platform that is commonly used as the means of validation for other high-throughput methods such as gene expression profiling (where mRNA expression does not always correlate with protein expression) or mass spectrometry (where initial detection is typically validated by subsequent MAb-based assays). Thus this platform combines the advantages of a high-throughput screen with a detection method that is sensitive and highly reproducible, as demonstrated by the high correlation coefficients obtained between replicate runs. Furthermore, the use of commercially available antibodies means that high quality reagents are immediately available for follow-up studies and/or development of more focused panels for specific applications such as 11-plex flow cytometry and/or CyTOF. Notably, only 5 out of 363 antibodies were found to be potentially problematic, in that they did not stain cell types that are reported to express the corresponding antigens (Table 1), supporting the robustness of this approach.

A requirement for FC analysis is that cells must be in single cell suspension, thus cultured adherent cells are detached by trypsinization and solid tumors are enzymatically dissociated. In addition it is common to use cryopreserved cells or to fix cells prior to analysis. We were concerned that these experimental manipulations would lead to significant changes in cell surface antigen detection. It is reassuring that detection of the majority of antigens assayed was stable; however detection of a number of markers was significantly altered. For the purposes of this study we have chosen to define a significant change as at least 5% absolute change *and* at least a two-fold change in relative detection. With that definition, eight antigens were significantly altered by fixation after staining, though most often to reduce the mean fluorescence intensity rather than the overall pattern of antigen detection. Nine antigens were altered by cryopreservation and thawing; one antigen, CD138 (syndecan-1) was reduced, while the remainder were increased (Table S5). The latter are almost exclusively expressed on lymphoid cells, and we hypothesize that these changes in apparent antigen detection may be accounted for by differential ability of different cell types in the pool to survive the cryopreservation and thawing process. Finally, 22 antigens were significantly altered by enzymatic digestion. All enzymes caused some change; trypsin, dispase and collagenase caused the most changes and are known proteases, but hyaluronidase and DNase also caused some alterations in antigen detection, though we cannot exclude impurity in the cell culture grade products used in routine practice in this setting. Of the 22 antigens showing significant change, the majority are reported to have extracellular proteolytic cleavage sites, which in some cases may be integral to the mechanism of action of the molecule (Table S3). While the majority of influenced antigens showed decrease with enzymatic digestion, occasional antigens demonstrated increased detection after enzymatic digestion, *e.g.* TWEAK/CD255 where a soluble form is known to be generated from the membrane-associated protein by cleavage [29]. While we validated each individual enzyme in isolation, in everyday practice these are often used in combination, *e.g.* collagenase and hyaluronidase in tissue digestion [30]. In the preliminary screen, some epitopes showed almost complete abrogation of detection, including common antigens such as CD1d, CD4, CD25, CD27 and CD62P, a particular concern for the study of heterogeneity and immunology in solid tumors. Of all the antigens detected in this platform CD138/syndecan-1 is the most labile, with alteration in detection upon cryopreservation and thawing, fixation, and collagenase digestion. CD138 is strongly expressed in myeloma but is also shed from the cell surface [31], as well as being sensitive to proteolytic cleavage during cell processing [32], thus we hypothesize that various tissue processing steps can increase this antigenic loss. Overall, these data show that the majority of cell surface antigens are not significantly affected by the various manipulations tested, but we provide a list of those that are altered as a resource to the research community. This illustrates the need to evaluate possible antigenic changes caused by experimental manipulations when new targets are being analyzed using flow cytometry methods.

Through the course of offering the HT-FC platform as a core facility resource at our institute, we have processed a large number of cell lines and other sample types. As shown in Figure 3, upon unsupervised hierarchical clustering of the percent-positive data the samples stratify naturally into clusters of related cell types. For example, all hematopoietic lineages form a major cluster, with lymphoid and myeloid leukemias and normal hematopoietic cells forming individual sub-clusters. The clustering of the CD45^+^ fraction of ccRCC samples with PBMCs nicely illustrates the consistent identification of a cell surface “signature” of hematopoietic cell lineages, even when derived from diverse sources (peripheral blood *vs*. surgically resected tumor samples). It also illustrates the consistent ability of the platform to identify a specific cell lineage, given that different samples were processed and analyzed by different individuals on different dates.

Epithelial cancer cell lines also form their own major cluster containing individual sub-clusters. Interestingly, the cell lines derived from a particular tumor type (*e.g.* ovarian cancer or head and neck cancer) do not perfectly segregate into type-specific clusters, but rather form sub-clusters that are intermingled with each other. The biological significance of this remains to be determined, but may relate to other properties such as the cell of origin of the individual cancer (*e.g.* stem cell or progenitor cell; ovarian surface epithelium or fallopian tube; primary tumor or metastasis; tongue *vs*. floor of mouth *vs*. other head and neck cancer sites; *etc*.), the mutational profile of the various cell lines, and/or the various culture conditions and selection pressures to which the cells have been exposed over the years. Notably, primary SOC samples form their own independent cluster, rather than clustering with ovarian cancer cell lines, suggesting an evolutionary divergence of the cell surface profile of passaged cell lines from that of the original primary tumors. It is also notable that cell lines in general express considerably fewer cell surface antigens overall than primary samples, suggesting that many cell surface proteins expressed *in vivo* are lost upon cell line establishment.

The other major cluster contained fibroblasts, and our in-house early passage ccRCC cell lines and primary ccRCC samples. In this case the cell lines did cluster with the primary samples, likely because they were in-house cell lines at early passage (passage 4 to 6), and thus perhaps had not yet diverged significantly in phenotype from the primary tumors from which they were derived. The clustering of ccRCC with fibroblasts is unexpected as ccRCC is considered to be an epithelial carcinoma. However, ccRCC has other evidence to suggest an intrinsically mesenchymal phenotype, such as its uniformly high expression of mesenchymal marker vimentin, lack of expression of epithelial structures such as cilia [33] and its propensity for differentiation along mesenchymal lineages as reported by Tun *et al* who demonstrated adipogenic and osteogenic transdifferentiation of ccRCC *in vitro* [34]. Thus our data provide further support to the concept that ccRCCs are phenotypically similar to other mesenchymal cell types, such as fibroblasts. Overall, our data suggests that the 363-marker cell surface profile is sufficient to molecularly define distinct cell types. This supports the utility of using the HT-FC platform for new lineage-specific marker discovery and molecular sub-classification of diseases, for biomarker discovery, and for research into biological mechanisms of disease. We are continuously expanding and refining the panel to include additional antibodies as they become available, and thus make the panel more comprehensive. The addition of more antibodies is done by simply adding new wells to the 96-well plates, and ultimately we may increase the plate number required from 4 to 5 as antibodies continue to become available. This does not significantly increase the cost or labor required to run the assay or analyse the data. We also have the flexibility to generate “custom plates” for those individuals who would prefer to assess a specific sub-panel of antibodies for a lower cost. Other innovations are also being developed, such as performing “bar coding” with intracellular fluorescent dyes that would allow multiple samples to be pooled and run simultaneously [26], thus increasing throughput and reducing costs even further.

One potential limitation of our assay is the approach of staining with one antibody at a time, rather than combining multiple antibodies together for multiplexing purposes. In some circumstances this is not the desired approach; however, the advantage of this format is that, as with other “array” type screening approaches, it can be applied to a wide range of cell types to rapidly provide a list of candidates, from which custom multiplexing panels can then be designed. This format also allows straightforward analysis of the expression profiles, and has the flexibility to allow co-staining with specific markers of interest to the individual investigator; thus multi-parameter information can still be obtained.

FC analysis of primary patient-derived tumor tissues can be used to identify cell subpopulations such as cancer-initiating cells, allowing their isolation for functional assays and molecular profiling. The process of identifying cell surface markers for such studies is challenging, and is often predicated on iterative examination of markers previously identified in other cancer or normal stem cell types. By co-staining with markers for various cell lineages present within primary tumors we were able to obtain marker profiles for the entire panel specifically on the cancer cell subset, providing a list of markers that are expressed on a small fraction of the cancer cells which might then be interrogated as markers of cell subpopulations, *e.g.* candidate cancer stem cell markers. Cell surface proteins are also excellent candidates for development of therapeutic antibodies, or biomarkers that can be prognostic, predictive of specific therapeutic responses, or used for disease detection and classification. Robust identification of markers in particular cellular subsets allows these hypotheses to be accurately examined. Furthermore, biomarkers may not be restricted only to the cancer cells; stromal cells such as fibroblasts, vascular endothelial cells and immune cells play important roles in tumor behavior and are even drug targets in some cases (*e.g.* vascular endothelial cells targeted by tyrosine-kinase inhibitors, the primary treatment modality for ccRCC patients with metastatic disease). The HT-FC platform allows for independent profiling of each individual population within the tumor for the entire 363 protein panel in a single assay, and thus represents a rapid and economical method for characterization of the cell populations present.

As with any high-throughput screening platform, it is essential to perform validation of candidates of interest that are identified with the screen. Follow-up studies should include MAb titration, and more standard in-tube staining and validation on larger cell numbers and/or additional samples. Here we present a case in which a marker discovered through the HT-FC screen was followed up and validated, and which will now provide a useful tool in the study of CAFs in SOC. CAFs are typically isolated by placing primary tumor samples into culture conditions in which the fibroblast population grows out, and can be passaged. However, this can introduce artifacts into analysis, as the CAFs may change their properties when no longer in the presence of cancer cells and/or may be influenced by the selection pressures of growing *in vitro*. A cell surface marker that would allow direct isolation of CAFs from tumor tissues by FACS would be extremely useful to facilitate their characterization. We found that surface markers reported for CAFs in other tumor types were not useful in our hands, so we performed HT-FC screening on cultured CAFs vs. primary SOC cells to find markers differentially expressed between the two. Several markers were found to be highly expressed on cultured CAFs and expressed at low levels on SOC cells, including CD90. When used in combination with epithelial cell marker EpCAM, a distinct population of EpCAM^−^/CD90^++^ cells was consistently observed. The use of CD90 is an improvement over using EpCAM alone and sorting the EpCAM^−^ subset, as there are EpCAM^−^ cancer cells present in some SOC samples, as previously determined by our group [35]. The addition of CD90 allowed the isolation of a population of cells that grows with the morphology of fibroblasts, expresses α-SMA, is negative for CK, and was shown to lack mutant p53 protein that was present in the matched cancer cells from the same patients. The latter verifies that the fibroblast population we are isolating does not arise as a result of epithelial-to-mesenchymal transition from the cancer cells, but represents a genetically normal stromal fibroblast population. CD90 was also found to be expressed to varying degrees on the EpCAM^+^ population, with a lower intensity of expression. While this did not hinder CAF isolation due the distinct population of EpCAM^−^CD90^++^ cells present even when EpCAM^+^ cells were CD90-positive, a more specific CAF marker would be useful. Several other markers were also identified in the screen (Figure 5), which can be tested for their ability to identify CAFs in SOC in the future.

## Conclusions

We have developed a high-throughput flow cytometry platform that can be used to rapidly profile the cell surfaceome, providing robust and reproducible data that can be used for a wide range of applications, including biomarker discovery, molecular classification of cancers, or identification of novel lineage specific or stem cell markers.

## Supporting Information

Figure S1 **Schematic of HT-FC workflow.**
(PDF)

Figure S2 **Gating strategy for HT-FC analysis of PBMCs.**
(PDF)

Figure S3 **Enlargement of dendrogram from heat map in** **Figure 3** **and principle components analysis of data from** **Figure 3** **.**
(PDF)

Figure S4 **Gating strategy for HT-FC analysis of immune cells, vascular endothelial cells, stromal fibroblasts, and cancer cells within primary ccRCC samples.**
(PDF)

Figure S5 **Gating strategy for FACS isolation of cancer-associated fibroblasts from primary serous ovarian cancer samples.**
(PDF)

Table S1 **Antibodies included in HT-FC panel.**
(PDF)

Table S2 **Validation of antigens influenced by enzymatic digestion.**
(PDF)

Table S3 **The 22 most-affected antigens appear predisposed to altered detection by enzymatic digestion.**
(PDF)

Table S4 **Fixation following antibody staining significantly alters detection of a small proportion of markers.**
(PDF)

Table S5 **Antigens with significant alteration in detection after cryopreservation and thawing.**
(PDF)

Table S6 **Cell surface profiling data of samples in** **Figure 3** **.**
(XLSX)

Table S7 **Clusters of antigens expressed on primary ccRCC cancer and stromal cell populations.**
(PDF)
