# Correction: Cell Surface Profiling Using High-Throughput Flow Cytometry: A Platform for Biomarker Discovery and Analysis of Cellular Heterogeneity

**DOI:** 10.1371/journal.pone.0114354

**Published:** 2014-11-25

**Authors:** The PLOS ONE Staff

Dr. Mona Meyer is not included in the author byline. She should be listed as the sixteenth author and affiliated with Hospital for Sick Children Research Institute, Toronto, ON, Canada. The contributions of this author are as follows: Contributed reagents/materials/analysis/tools.
