# Supplementary material for: Cell Surface Profiling Using High-Throughput Flow Cytometry: A Platform for Biomarker Discovery and Analysis of Cellular Heterogeneity
Source: PLoS One. 2014 Aug 29;9(8):e105602. doi: 10.1371/journal.pone.0105602 (PMC4149490; doi:10.1371/journal.pone.0105602)
Supplement: Figure S1 — Schematic of HT-FC workflow. (PDF) [file pone.0105602.s001.pdf]

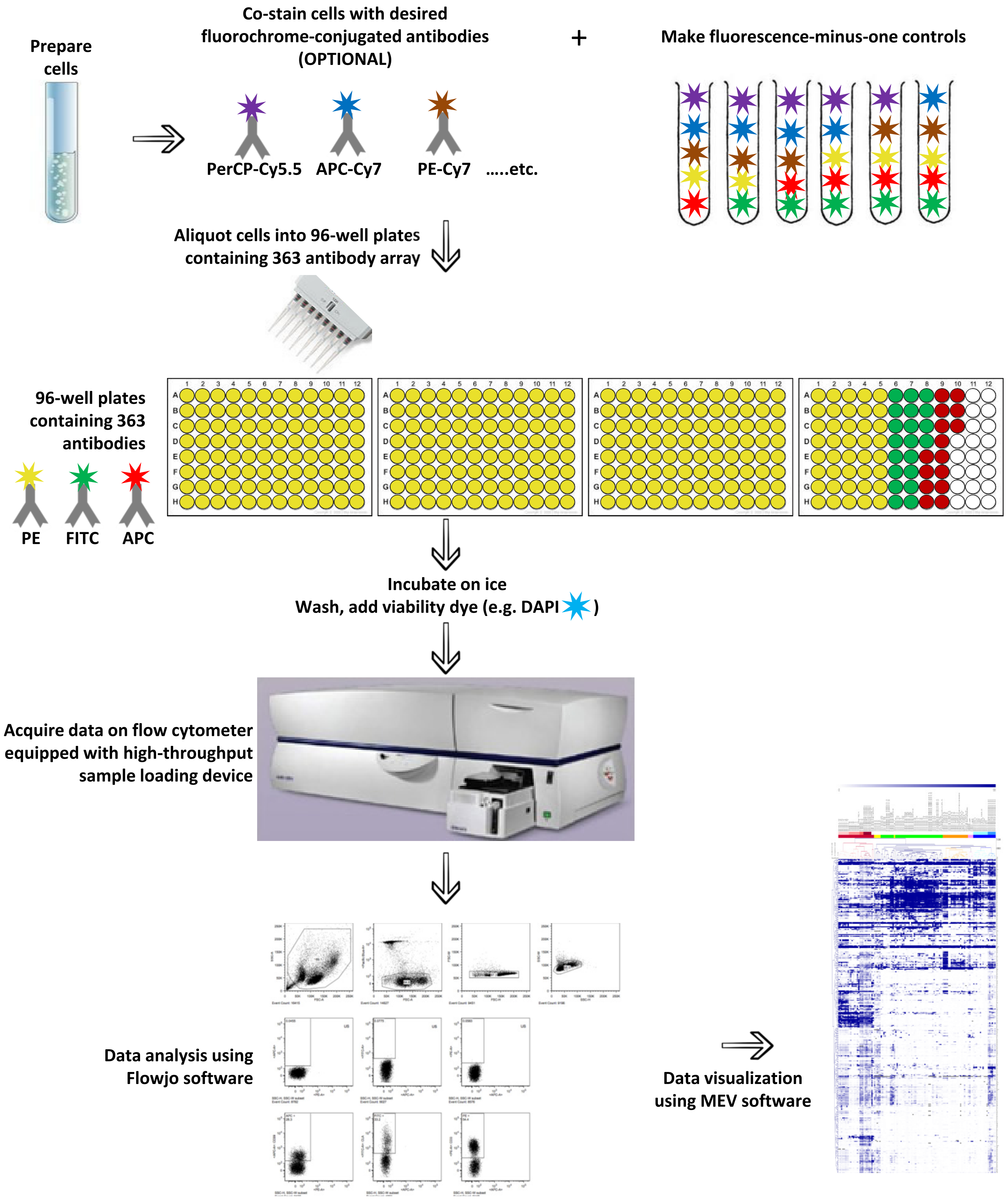

Figure S1: Schematic of HT-FC workflow. Prepared cells are initially co-stained, if desired, with antibodies conjugated to fluorochromes that do not overlap with those in the HT-FC panel (optional). Fluorescence-minus-one controls are made for each fluorochrome-conjugated antibody. If no co-staining antibodies are used, fluorescence-minus-one controls for the antibodies in the HT-FC panel can be made in the plates by adding cells to wells with no antibodies in them. After data acquisition, data is analyzed as illustrated in Figures S2 (no co-staining) and S4 (with co-staining), and as described in the manuscript.
