# Supplementary material for: Cell Surface Profiling Using High-Throughput Flow Cytometry: A Platform for Biomarker Discovery and Analysis of Cellular Heterogeneity
Source: PLoS One. 2014 Aug 29;9(8):e105602. doi: 10.1371/journal.pone.0105602 (PMC4149490; doi:10.1371/journal.pone.0105602)
Supplement: Figure S2 — Gating strategy for HT-FC analysis of PBMCs. (PDF) [file pone.0105602.s002.pdf]

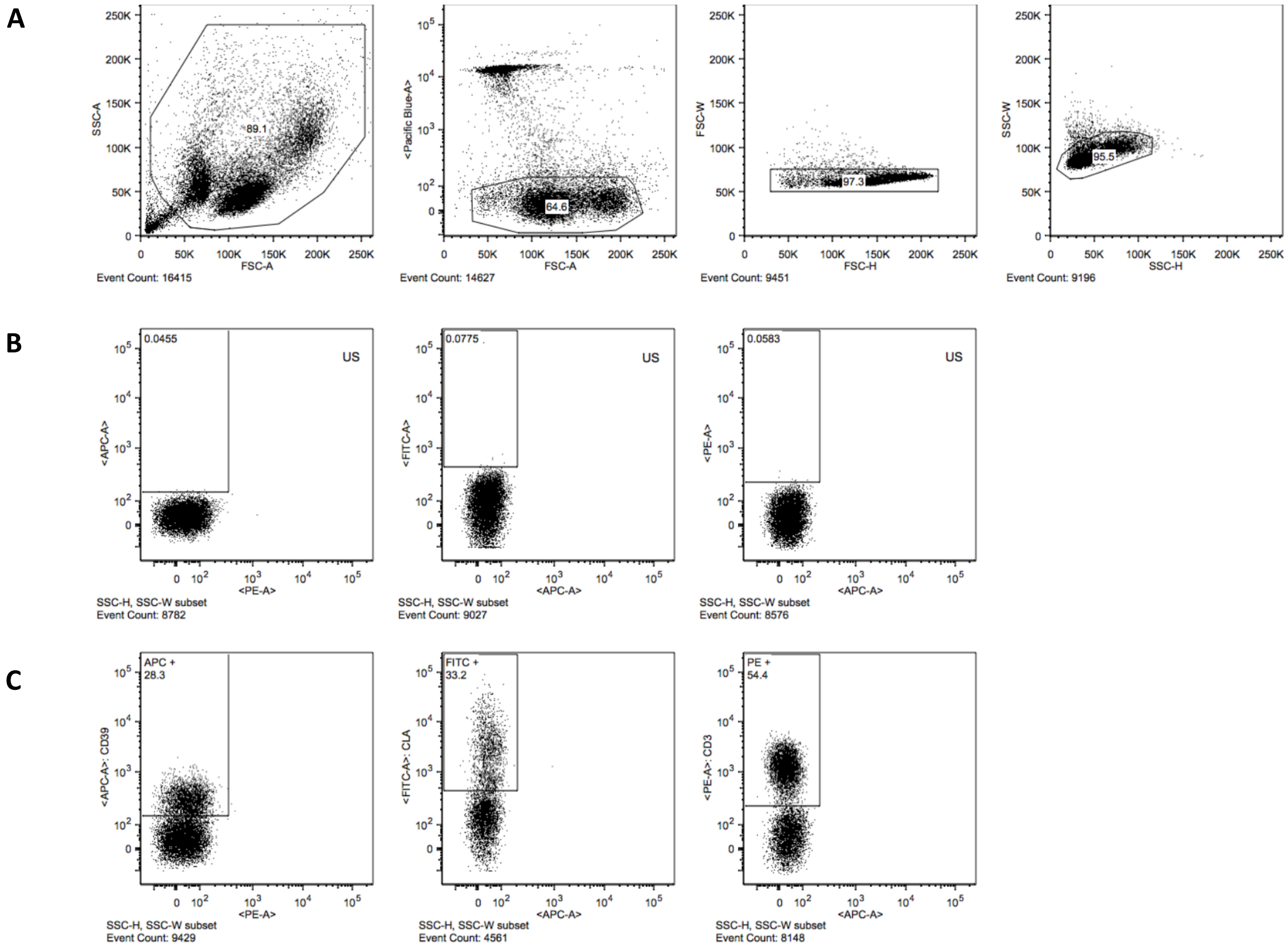

Figure S2. Gating strategy for HT-FC analysis of PBMCs. (A) Cells were gated on forward scatter (FSC) *vs*. side scatter (SCC) to exclude debris and then on DAPI-negative viable cells, and doublets were excluded using FSC and SSC height-*vs.*-width plots. (B) Fluorescence-minus-one controls for each of the three fluorochromes in the HT-FC panel are shown. (C) Examples of positively expressed markers for each of the three fluorochromes used are shown.
