# Supplementary material for: Cell Surface Profiling Using High-Throughput Flow Cytometry: A Platform for Biomarker Discovery and Analysis of Cellular Heterogeneity
Source: PLoS One. 2014 Aug 29;9(8):e105602. doi: 10.1371/journal.pone.0105602 (PMC4149490; doi:10.1371/journal.pone.0105602)
Supplement: Figure S3 — Enlargement of dendrogram from heat map in Figure 3 and principle components analysis of data from Figure 3 . (PDF) [file pone.0105602.s003.pdf]

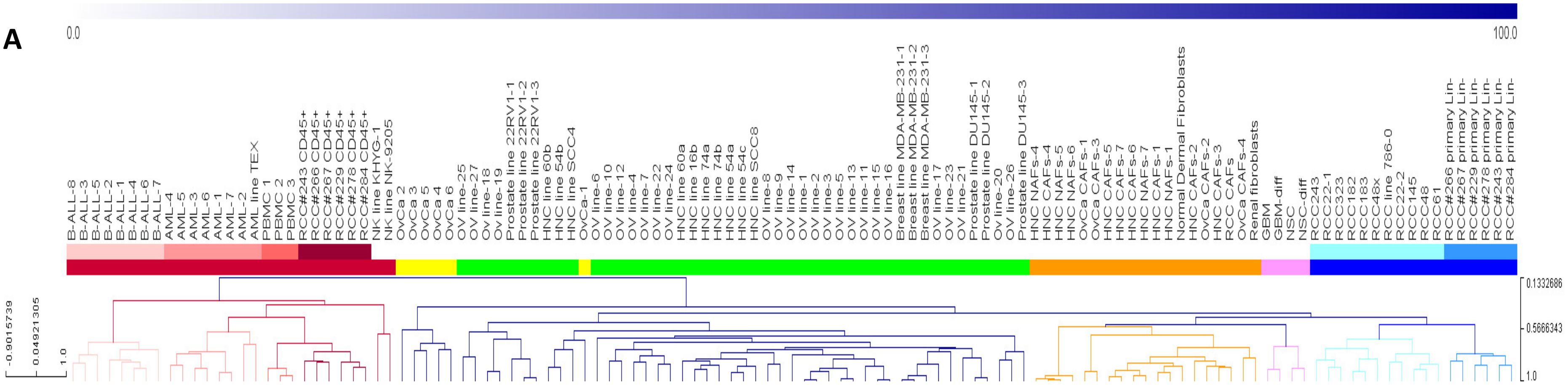

Red: hematopoietic
Yellow: primary ovarian cancer samples
Green: epithelial cancer cell lines
Orange: fibroblasts
Pink: normal neural and GBM stem cells
Blue: ccRCC cell lines and primary samples

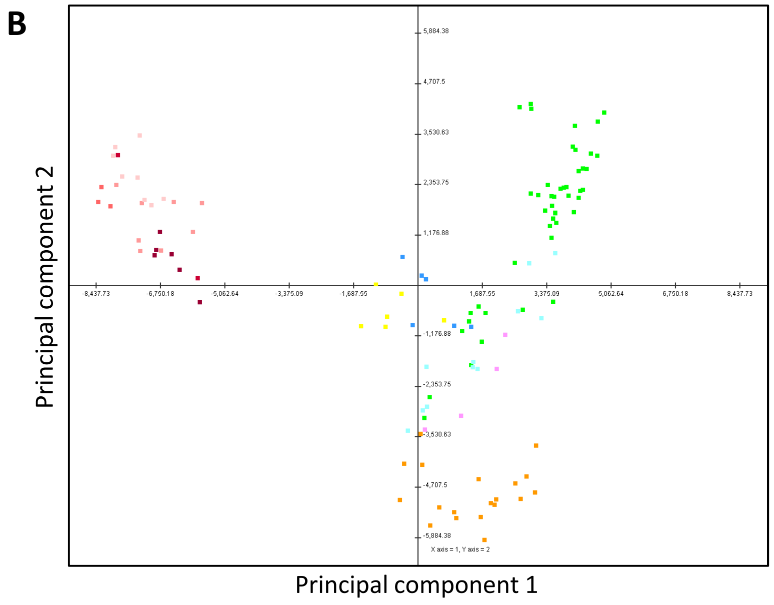

Figure S3. (A) Enlargement of dendrogram from heat map in Figure 3. Unsupervised hierarchical clustering of %+ marker expression profiles was performed. B-ALL: B cell acute lymphocytic leukemia; AML: acute myeloid leukemia; NK: natural killer cell; PBMC: peripheral blood mononuclear cells; RCC: renal cell carcinoma; OvCa: primary ovarian cancer samples; OV line: ovarian cancer cell line; HNC line: head and neck cancer cell line; CAFs: cancer-associated fibroblasts; NAFs: normal adjacent fibroblasts; GBM: glioblastoma multiforme; diff: differentiating culture conditions; NSC: neuronal stem cells. **(B)** Principle components analysis of data from (A).
