# Supplementary material for: Cell Surface Profiling Using High-Throughput Flow Cytometry: A Platform for Biomarker Discovery and Analysis of Cellular Heterogeneity
Source: PLoS One. 2014 Aug 29;9(8):e105602. doi: 10.1371/journal.pone.0105602 (PMC4149490; doi:10.1371/journal.pone.0105602)
Supplement: Figure S4 — Gating strategy for HT-FC analysis of immune cells, vascular endothelial cells, stromal fibroblasts, and cancer cells within primary ccRCC samples. (PDF) [file pone.0105602.s004.pdf]

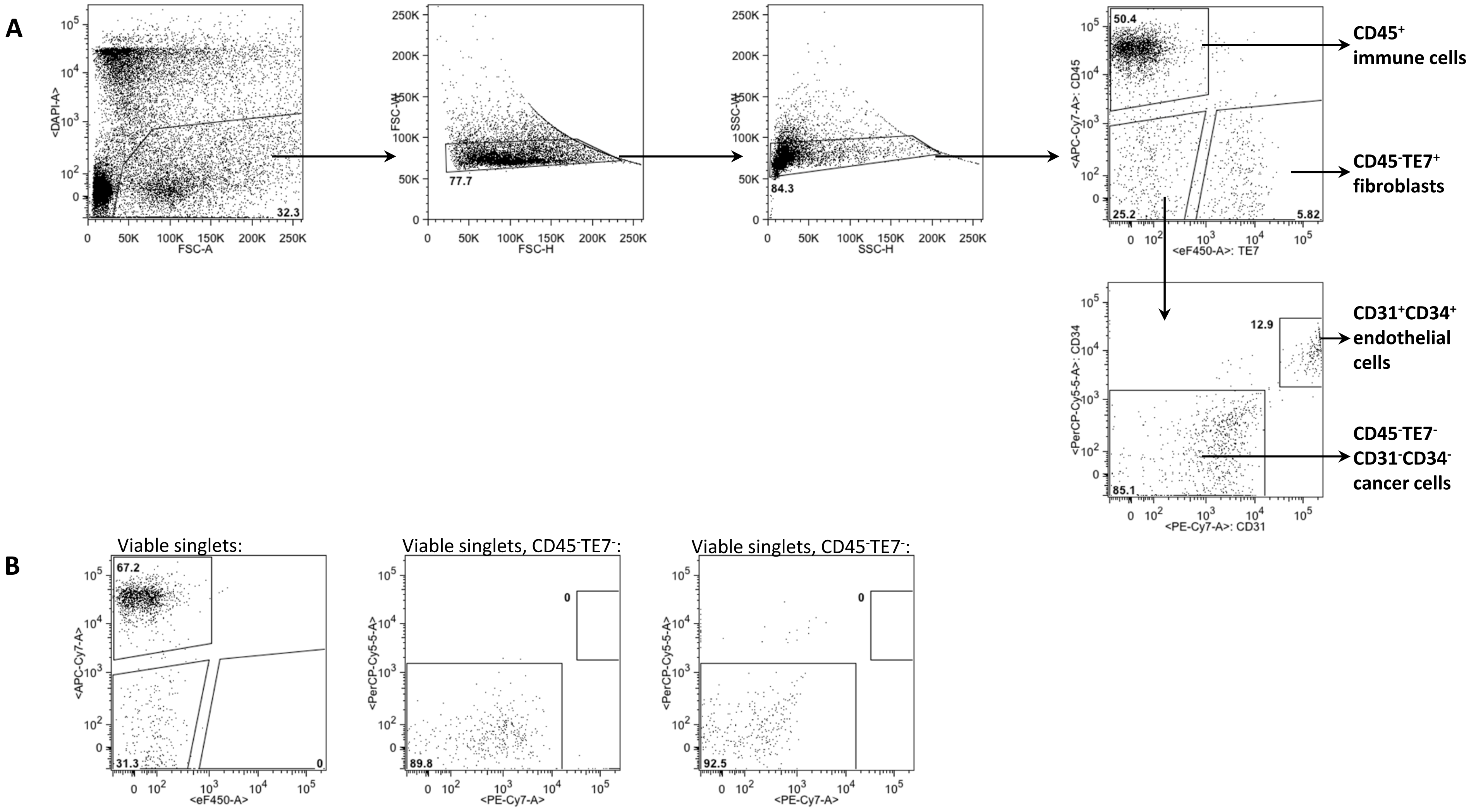

Figure S4. Gating strategy for HT-FC analysis of immune cells, vascular endothelial cells, stromal fibroblasts, and cancer cells within primary ccRCC samples. (A) Cells were gated on DAPI-negative viable cells and doublets were excluded using FSC and SSC height-*vs.*-width plots. Gates were then established for CD45+ immune cells and TE7+ fibroblasts. CD45-TE7- cells were further gated for CD31+CD34+ vascular endothelial cells, and CD31-CD34- cancer cells. (B) Fluorescence-minus-one controls for TE7-eF450 (left), CD31-PE-Cy7 (middle) and CD34-PerCP-Cy5.5 (right) are shown, with the same gates applied as in (A).
