# Supplementary material for: Cell Surface Profiling Using High-Throughput Flow Cytometry: A Platform for Biomarker Discovery and Analysis of Cellular Heterogeneity
Source: PLoS One. 2014 Aug 29;9(8):e105602. doi: 10.1371/journal.pone.0105602 (PMC4149490; doi:10.1371/journal.pone.0105602)
Supplement: Figure S5 — Gating strategy for FACS isolation of cancer-associated fibroblasts from primary serous ovarian cancer samples. (PDF) [file pone.0105602.s005.pdf]

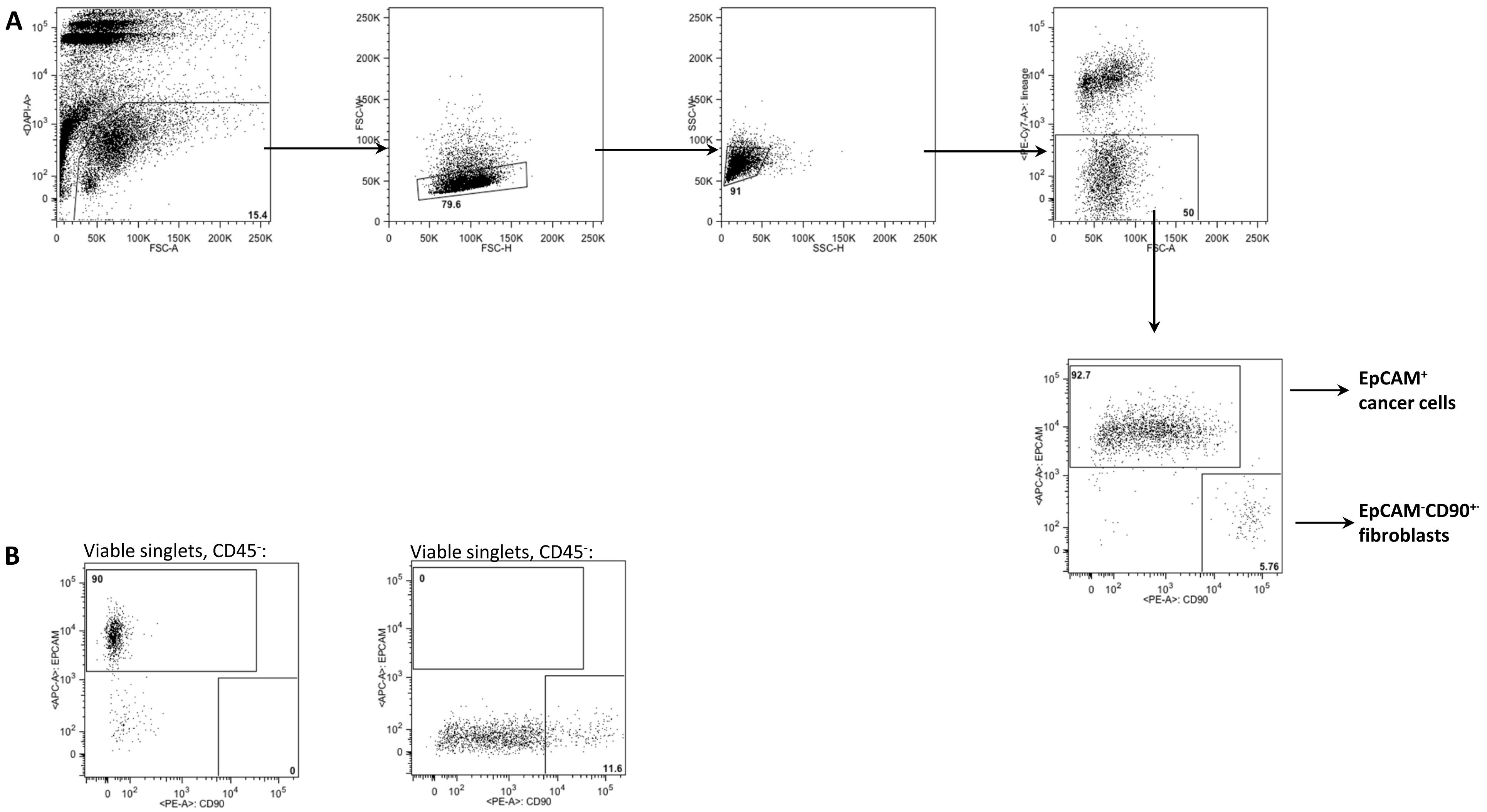

Figure S5. Gating strategy for FACS isolation of cancer-associated fibroblasts from primary serous ovarian cancer samples. (A) Cells were gated on DAPI-negative viable cells and doublets were excluded using FSC and SSC height-*vs.*-width plots. CD45+/CD31+ "lineage postive" immune and vascular endothelial cells were excluded, and cells were further gated for EpCAM+ cancer cells, and EpCAM-CD90++ fibroblasts. (B) fluorescence-minus-one controls for CD90-PE (left) and EpCAM-APC (right) are shown, with the same gates applied as in (A).
