# Supplementary material for: Cell Surface Profiling Using High-Throughput Flow Cytometry: A Platform for Biomarker Discovery and Analysis of Cellular Heterogeneity
Source: PLoS One. 2014 Aug 29;9(8):e105602. doi: 10.1371/journal.pone.0105602 (PMC4149490; doi:10.1371/journal.pone.0105602)
Supplement: Table S1 — Antibodies included in HT-FC panel. (PDF) [file pone.0105602.s006.pdf]

Table S1. Antibodies included in HT-FC panel.

Antigen	Isotype	NCBI Gene Name	Entrez Gene ID	Supplier	Clone	Note
BLTR-1	Mouse IgG1	LTB4R	1241	BD Biosciences	203/14F11	
CA9	Mouse IgG2a	CA9	768	R&D Systems	303123	
CD10	Mouse IgG1, κ	MME	4311	BD Biosciences	HI10a	
CD100	Mouse IgM	SEMA4D	10507	e-Bioscience	133-1C6	
CD101	Mouse IgG1	IGSF2	9398	e-Bioscience	BB27	
CD102	Mouse IgG2a, κ	ICAM2	3384	BD Biosciences	CBR-1C2/2.1	
CD103	Mouse IgG1, κ	ITGAE	3682	BD Biosciences	Ber-ACT8	
CD104	Rat IgG2b, κ	ITGB4	3691	BD Biosciences	439-9B	
CD105	Mouse IgG1	ENG	2022	e-Bioscience	SN6	
CD106	Mouse IgG1, κ	VCAM1	7412	BD Biosciences	51-10C9	
CD107a	Mouse IgG1, κ	LAMP1	3916	BD Biosciences	H4A3	
CD107b	Mouse IgG1, κ	LAMP2	3920	BD Biosciences	H4B4	
CD108	Mouse IgG2a, κ	SEMA7A	8482	BD Biosciences	KS-2	
CD109	Mouse IgG1, κ	CD109	135228	BD Biosciences	TEA 2/16	
CD110	Mouse IgG2b, κ	MPL	4352	BD Biosciences	1.6.1	
CD111	Mouse IgG1, κ	PVRL1	5818	BioLegend	R1.302	
CD112	Mouse IgG1, κ	PVRL2	5819	BD Biosciences	R2.525	
CD114	Mouse IgG1, κ	CSF3R	1441	BD Biosciences	LMM741	
CD115	Mouse IgG1	CSF1R	1436	R&D Systems	61708	
CD116	Mouse IgG1, κ	CSF2RA	1438	BD Biosciences	hGMCSFR-M1	
CD117	Mouse IgG1, κ	KIT	3815	BD Biosciences	YB5.B8	
CD118	Mouse IgG1	LIFR	3977	R&D Systems	32953	
CD119	Mouse IgG1, κ	IFNGR1	3459	BD Biosciences	GIR-208	
CD11a	Mouse IgG1, κ	ITGAL	3683	BD Biosciences	HI111	
CD11b	Mouse IgG1, κ	ITGAM	3684	BD Biosciences	ICRF44	
CD11c	Mouse IgG1, κ	ITGAX	3687	BD Biosciences	B-ly6	
CD120a	Mouse IgG1	TNFRSF1A	7132	R&D Systems	16803	
CD120b	Rat IgG2b, κ	TNFRSF1B	7133	BD Biosciences	hTNFR-M1	
CD121b	Mouse IgG1	IL1R2	7850	R&D Systems	34141	
CD122	Mouse IgG1, κ	IL2RB	3560	BD Biosciences	Mik-β3	
CD123	Mouse IgG2a, κ	IL3RA	3563	BD Biosciences	7G3	
CD124	Mouse IgG1, κ	IL4R	3566	BD Biosciences	hIL4R-M57	
CD125	Mouse IgG1	IL5RA	3568	R&D Systems	26815	
CD126	Mouse IgG1, κ	IL6R	3570	BD Biosciences	M5	
CD127	Mouse IgG1, κ	IL7R	3575	BD Biosciences	hIL-7R-M21	
CD129	Mouse IgG2b, k	IL9R	3581	BioLegend	AH9R7	
CD13	Mouse IgG1, κ	ANPEP	290	BD Biosciences	WM15	
CD130	Mouse IgG1, κ	IL6ST	3572	BD Biosciences	AM64	
CD131	Mouse IgG1, κ	CSF2RB	1439	e-Bioscience	1C1	
CD132	Mouse IgG1, κ	IL2RG	3561	BD Biosciences	AG184	
CD133	Mouse IgG1, κ	PROM1	8842	Miltenyi	AC133	
CD134	Mouse IgG1, κ	TNFRSF4	7293	BD Biosciences	ACT35	
CD135	Mouse IgG1, κ	FLT3	2322	BD Biosciences	4G8	
CD136	not given	MST1R	4486	Beckman Coulter	ID1	
CD137	Mouse IgG1, κ	TNFRSF9	3604	BD Biosciences	4B4-1	
CD137L	Mouse IgG1, κ	TNFSF9	8744	BioLegend	5F4	
CD138	Mouse IgG1, κ	SDC1	6382	BD Biosciences	MI15	
CD14	Mouse IgG2a, κ	CD14	929	BD Biosciences	M5E2	
CD140a	Mouse IgG2a, κ	PDGFRA	5156	BD Biosciences	αR1	
CD140b	Mouse IgG2a, κ	PDGFRB	5159	BD Biosciences	28D4	
CD141	Mouse IgG1, κ	THBD	7056	BD Biosciences	1A4	
CD142	Mouse IgG1, κ	F3	2152	BD Biosciences	HTF-1	
CD143	Mouse IgG1	ACE	1636	R&D Systems	171417	
CD144	Mouse IgG1, κ	CDH5	1003	BD Biosciences	55-7H1	
CD146	Mouse IgG1, κ	MCAM	4162	BD Biosciences	P1H12	
CD147	Mouse IgG1, κ	BSG	682	BD Biosciences	HIM6	
CD148	Mouse IgG1	PTPRJ	5795	R&D Systems	143-41	
CD15	Mouse IgM, k	FUT4	2526	BD Biosciences	HI98	
CD150	Mouse IgG1, κ	SLAMF1	6504	BD Biosciences	A12	
CD151	Mouse IgG1, κ	CD151	977	BD Biosciences	14A2.H1	
CD152	Mouse IgG2a, κ	CTLA4	1493	BD Biosciences	BNI3	
CD153	Mouse IgG2B	TNFSF8	944	R&D Systems	116614	
CD154	Mouse IgG1, κ	CD40LG	959	BD Biosciences	TRAP1	
CD155	Mouse IgG1	PVR	5817	e-Bioscience	2H7CD155	

Supplementary Table 1. Antibodies included in HT-FC panel, continued.

Antigen	Isotype	NCBI Gene Name	Entrez Gene ID	Supplier	Clone	Note
CD156b	Mouse IgG1	ADAM17	6868	R&D Systems	111633	
CD157	Mouse IgG1, κ	BST1	683	MBL	RF3	
CD158A	Mouse IgM, κ	KIR2DL1	3802	BD Biosciences	HP-3E4	
CD158B1	Mouse IgG2b, k	KIR2DL2	3803	BD Biosciences	CH-L	
CD158B2	Mouse IgG2a, κ	KIR2DL3	3804	BD Biosciences	DX27	
CD158D	Mouse IgG2a	KIR2DL4	3805	R&D Systems	181703	
CD158E2	Mouse IgG1, κ	KIR3DS1	3813	BD Biosciences	DX9	
CD158F	Mouse IgG1	KIR2DL5A	57292	BioLegend	UP-R1	
CD158I	Mouse IgG1	KIR2DS4	3809	Miltenyi	JJC11.6	
CD159a	Mouse IgG2a	KLRC1	3821	R&D Systems	131411	
CD159c	Mouse IgG1	KLRC2	3822	R&D Systems	134591	
CD16	Mouse IgG1, κ	FCGR3A	2214	BD Biosciences	3G8	
CD160	Mouse IgM, κ	CD160	11126	BioLegend	BY55	
CD161	Mouse IgG1, κ	KLRB1	3820	BD Biosciences	DX12	
CD162	Mouse IgG1, κ	SELPLG	6404	BD Biosciences	KPL-1	
CD163	Mouse IgG1, κ	CD163	9332	BD Biosciences	GHI/61	
CD164	Mouse IgG2a, κ	CD164	8763	BD Biosciences	N6B6	
CD165	Mouse IgG1	CD165	23449	e-Bioscience	SN2 N56-D11	
CD166	Mouse IgG1, κ	ALCAM	214	BD Biosciences	3A6	
CD167a	Mouse IgM, k	DDR1	780	BioLegend	51D6	
CD169	Mouse IgG1	SIGLEC1	6614	Biolegend	7-239	
CD16b	Mouse IgG2a, κ	FCGR3B	2215	BD Biosciences	CLB-gran11.5	
CD17	Mouse IgM	N/A	N/A	Lifespan Bioscienc	Not given	Lactosylceramide
CD170	Mouse IgG1	SIGLEC5	8778	R&D Systems	194128	
CD171	Mouse IgG2a	L1CAM	3897	e-Bioscience	5G3	
CD172a	Mouse IgG1, κ	SIRPA	140885	BioLegend	SE5A5	
CD172b	Mouse IgG1, κ	SIRPB1	10326	BD Biosciences	B4B6	
CD172g	Mouse IgG1, κ	SIRPG	55423	BioLegend	LSB2.20	
CD175s	Mouse IgG1	N/A	N/A	Abcam	STn 219	Sialyl-Tn
CD177	Mouse IgG1	CD177	57126	Abcam	MEM-166	
CD178	Mouse IgG1, κ	FASLG	356	BioLegend	NOK-1	
CD179a	Mouse IgG1, κ	VPREB1	7441	BioLegend	HSL96	
CD18	Mouse IgG1, κ	ITGB2	3689	BD Biosciences	6.7	
CD180	Mouse IgG1, κ	CD180	4064	BD Biosciences	G28-8	
CD181	Mouse IgG2b, k	IL8RA	3577	BD Biosciences	5A12	
CD182	Mouse IgG1, κ	IL8RB	3579	BD Biosciences	6C6	
CD183	Mouse IgG1, κ	CXCR3	2833	BD Biosciences	1C6/CXCR3	
CD184	Mouse IgG2a, κ	CXCR4	7852	BD Biosciences	12G5	
CD185	Mouse IgG2b, k	BLR1	643	BD Biosciences	RF8B2	
CD186	Mouse IgG2b, k	CXCR6	10663	R&D Systems	56811	
CD19	Mouse IgG1, κ	CD19	930	BD Biosciences	HIB19	
CD191	Mouse IgG2b	CCR1	1230	R&D Systems	53504	
CD192	Mouse IgG2b, k	CCR2	729230	BD Biosciences	48607	
CD193	Mouse IgG2b, k	CCR3	1232	BD Biosciences	5E8	
CD194	Mouse IgG2b, k	CCR4	1233	BioLegend	TG6/CCR4	
CD195	Mouse IgG2a, κ	CCR5	1234	BD Biosciences	3A9	
CD196	Mouse IgG1, κ	CCR6	1235	BD Biosciences	11A9	
CD197	Rat IgG2a, κ	CCR7	1236	BD Biosciences	3D12	
CD1a	Mouse IgG1, κ	CD1A	909	BD Biosciences	HI149	
CD1b	Mouse IgG1, κ	CD1B	910	BD Biosciences	M-T101	
CD1c	Mouse IgG1, κ	CD1C	911	BioLegend	L161	
CD1d	Mouse IgG1, κ	CD1D	912	BD Biosciences	CD1d42	
CD2	Mouse IgG1, κ	CD2	914	BD Biosciences	RPA-2.10	
CD20	Mouse IgG2b, k	MS4A1	931	BD Biosciences	2H7	
CD200	Mouse IgG1, κ	CD200	4345	BD Biosciences	MRC OX-104	
CD201	Rat IgG1, k	PROCR	10544	BD Biosciences	RCR-252	
CD202b	Mouse IgG1, κ	TEK	7010	BioLegend	33.1 (Ab33)	
CD203c	Mouse IgG1, κ	ENPP3	5169	BioLegend	NP4D6	
CD204	Mouse IgG2B	MSR1	4481	R&D Systems	351615	
CD205	Mouse IgG2b, k	LY75	4065	BD Biosciences	MG38	
CD206	Mouse IgG1, κ	MRC1	4360	BD Biosciences	19.2	
CD207	Mouse IgG1	CD207	50489	R&D Systems	343828	
CD208	Mouse IgG1, κ	LAMP3	27074	BD Biosciences	I10-1112	
CD209	Mouse IgG2b, k	CD209	30835	BD Biosciences	DCN46	
CD21	Mouse IgG1, κ	CR2	1380	BD Biosciences	B-ly4	

Supplementary Table 1. Antibodies included in HT-FC panel, continued.

Antigen	Isotype	NCBI Gene Name	Entrez Gene ID	Supplier	Clone	Note
CD212	Mouse IgG1, κ	IL12RB1	3594	BD Biosciences	2.4e6	
CD213a2	Mouse IgG1	IL13RA2	3598	Abcam	B-D13	
CD215	Mouse IgG2B	IL15RA	3601	R&D Systems	151303	
CD217	Mouse IgG1	IL17RA	23765	bioLegend	BG/hIL17AR	
CD218b	Mouse IgG2b	IL18RAP	8807	R&D Systems	132029	
CD22	Mouse IgG2b, k	CD22	933	BD Biosciences	S-HCL-1	
CD220	Goat IgG	INSR	3643	R&D Systems		Polyclonal
CD221	Mouse IgG1, κ	IGF1R	3480	BD Biosciences	1H7	
CD222	Mouse IgG1, κ	IGF2R	3482	BioLegend	MEM-238	
CD223	Goat IgG	LAG3	3902	R&D Systems	not given	
CD226	Mouse IgG1, κ	CD226	10666	BD Biosciences	DX11	
CD227	Mouse IgG1, κ	MUC1	4582	BD Biosciences	HMPV	
CD229	Mouse IgG2a	LY9	4063	R&D Systems	249936	
CD23	Mouse IgG1, κ	FCER2	2208	BD Biosciences	M-L233	
CD230	Mouse IgG1, κ	PRNP	5621	e-Bioscience	4D5	
CD231	Mouse IgG1, κ	TSPAN7	7102	BioLegend	SN1a (M3-3D9)	
CD234	Mouse IgG2A	DARC	2532	R&D Systems	358307	
CD235a	Mouse IgG2b, k	GYPA	2993	BD Biosciences	GA-R2 (HIR2)	
CD24	Mouse IgG2a, κ	CD24	100133941	BD Biosciences	ML5	
CD243 (BC)	Mouse IgG2a	ABCB1	5243	Beckman Coulter	UIC2	
CD243 (BD)	Mouse IgG2b, k	ABCB1	5243	BD Biosciences	17F9	
CD244	Mouse IgG2a, κ	CD244	51744	BD Biosciences	2-69	
CD245	Mouse IgG1, κ	NPAT	4863	BioLegend	DY12	
CD249	Rat IgG1, k	ENPEP	2028	Lifespan Bioscienc	not provided	
CD25	Mouse IgG1, κ	IL2RA	3559	BD Biosciences	M-A251	
CD252	Mouse IgG1, κ	TNFSF4	7292	BD Biosciences	Ik-1	
CD253	Mouse IgG1	TNFSF10	8743	BD Biosciences	RIK-2	
CD254	Mouse IgG2b, k	TNFSF11	8600	BioLegend	MIH24	
CD255	Mouse IgG3	TNFSF12	8742	BD Biosciences	CARL-1	
CD256	Mouse IgG2a, κ	TNFSF13	8741	BioLegend	A3D8	
CD257	Mouse IgG1, κ	TNFSF13B	10673	BioLegend	T7-241	
CD258	Mouse IgG1	TNFSF14	8740	R&D Systems	115520	
CD26	Mouse IgG1, κ	DPP4	1803	BD Biosciences	M-A261	
CD261	Mouse IgG1	TNFRSF10A	8797	BioLegend	DJR1	
CD262	Mouse IgG2b	TNFRSF10B	8795	R&D Systems	71908	
CD263	Mouse IgG1	TNFRSF10C	8794	R&D Systems	90906	
CD264	Mouse IgG1	TNFRSF10D	8793	R&D Systems	104918	
CD267	Rat IgG2a, κ	TNFRSF13B	23495	BD Biosciences	1A1-K21-M22	
CD268	Mouse IgG1, κ	TNFRSF13C	115650	BD Biosciences	11C1	
CD269	Goat IgG	TNFRSF17	608	R&D Systems	Polyclonal	
CD27	Mouse IgG1, κ	CD27	939	BD Biosciences	M-T271	
CD270	Mouse IgG1, κ	TNFRSF14	8764	BioLegend	122	
CD271	Mouse IgG1, κ	NGFR	4804	BD Biosciences	C40-1457	
CD272	Mouse IgG1, κ	BTLA	151888	BD Biosciences	J168-540.90.22	
CD273	Mouse IgG1, κ	PDCD1LG2	80380	BD Biosciences	MIH18	
CD274	Mouse IgG1, κ	CD274	29126	BD Biosciences	MIH1	
CD275	Mouse IgG2b, k	ICOSLG	23308	BD Biosciences	2D3/B7-H2	
CD276	Mouse IgG1, κ	CD276	80381	BioLegend	DCN.70	
CD277	Mouse IgG1	BTN3A1	11119	e-Bioscience	BT3.1	
CD278	Mouse IgG1	ICOS	29851	BD Biosciences	DX29	
CD279	Mouse IgG1, κ	PDCD1	5133	BD Biosciences	MIH4	
CD28	Mouse IgG1, κ	CD28	940	BD Biosciences	CD28.2	
CD281	Mouse IgG1, κ	TLR1	7096	BioLegend	TLR1.136	
CD282	Mouse IgG1, κ	TLR2	7097	BD Biosciences	11G7	
CD283	Mouse IgG1, κ	TLR3	7098	e-Bioscience	TLR3.7	
CD284	Mouse IgG2a	TLR4	7099	R&D Systems	610015	
CD286	Mouse IgG1, κ	TLR6	10333	BioLegend	TLR6.127	
CD288	Mouse IgG1	TLR8	51311	Abcam	44C143	
CD289	Rat IgG2a, κ	TLR9	54106	BD Biosciences	eB72-1665	
CD29	Mouse IgG1, κ	ITGB1	3688	BD Biosciences	MAR4	
CD290	Mouse IgG1, κ	TLR10	81793	BioLegend	3C10C5	
CD292	Goat IgG	BMPR1A	657	R&D Systems	Polyclonal	
CD294	Rat IgG2a, κ	GPR44	11251	BD Biosciences	BM16	
CD295	Mouse IgG2b	LEPR	3953	R&D Systems	52263	
CD298	Mouse IgG2a	ATP1B3	483	BioLegend	LNH-94	

Supplementary Table 1. Antibodies included in HT-FC panel, continued.

Antigen	Isotype	NCBI Gene Name	Entrez Gene ID	Supplier	Clone	Note
CD299	Mouse IgG2b	CLEC4M	10332	R&D Systems	120604	
CD3	Mouse IgG2a, κ	CD3E	916	BD Biosciences	HIT3a	
CD30	Mouse IgG1, κ	TNFRSF8	943	BD Biosciences	BerH8	
CD300a	Mouse IgG1	CD300A	11314	Abcam	MEM-260	
CD300c	Mouse IgG1, κ	CD300C	10871	BioLegend	TX45	
CD300e	Mouse IgG1, κ	CD300E	342510	BioLegend	UP-H2	
CD301	Mouse IgG1	CLEC10A	10462	Imgenex	125A10.03	
CD303	Mouse IgG1	CLEC4C	170482	Miltenyi	AC144	
CD304	Mouse IgG1	NRP1	8829	Miltenyi	AD5-17F6	
CD305	Mouse IgG1, κ	LAIR1	3903	BD Biosciences	DX26	
CD307e	Mouse IgG2a, κ	FCRL5	83416	BioLegend	509f6	
CD309	Mouse IgG1	KDR	3791	R&D Systems	89106	
CD31	Mouse IgG1, κ	PECAM1	5175	BD Biosciences	WM59	
CD312	Mouse IgG1	EMR2	30817	AbD Serotech	2A1	
CD314	Mouse IgG1, κ	KLRK1	22914	BD Biosciences	1D11	
CD317	Mouse IgG1, κ	BST2	684	BioLegend	RS38E	
CD318	Mouse IgG2a	CDCP1	64866	R&D Systems	309121	
CD319	Mouse IgG2a	SLAMF7	57823	R&D Systems	235614	
CD32	Mouse IgG1, κ	FCGR2A	2212	BD Biosciences	3D3	
CD321	Mouse IgG1, κ	F11R	50848	BD Biosciences	M.AB.F11	
CD322	Rat IgG2a	JAM2	58494	AbD Serotech	CRAM-18 F26	
CD324	Mouse IgG2a, κ	CDH1	999	BD Biosciences	67A4	
CD325	Mouse IgG1, κ	CDH2	1000	e-Bioscience	8C11	
CD326	Mouse IgG1, κ	TACSTD1	4072	BD Biosciences	EBA-1	
CD328	Mouse IgG1, κ	SIGLEC7	27036	BD Biosciences	F023-420	
CD33	Mouse IgG1, κ	CD33	945	BD Biosciences	P67.6	
CD332	Mouse IgG1	FGFR2	2263	R&D Systems	98725	
CD333	Mouse IgG1	FGFR3	2261	R&D Systems	136334	
CD334	Mouse IgG1, κ	FGFR4	2264	BioLegend	4FR6D3	
CD335	Mouse IgG1, κ	NCR1	9437	BD Biosciences	9E2/NKp46	
CD336	Mouse IgG1, κ	NCR2	9436	BD Biosciences	P44-8.1	
CD337	Mouse IgG1, κ	NCR3	259197	BD Biosciences	P30-15	
CD338	Mouse IgG2b, k	ABCG2	9429	BioLegend	5D3	
CD339	Mouse IgG2b	JAG1	182	R&D Systems	188331	
CD34	Mouse IgG1, κ	CD34	947	BD Biosciences	581	
CD340	Mouse IgG1, κ	ERBB2	2064	BD Biosciences	Neu 24.7	
CD344	Mouse IgG1, κ	FZD4	8322	BioLegend	CH3A4A7	
CD349	Mouse IgM, k	FZD9	8326	BioLegend	W3C4E11	
CD35	Mouse IgG1, κ	CR1	1378	BD Biosciences	E11	
CD351	Mouse IgG1, κ	FCAMR	83953	BioLegend	TX61	
CD352	Mouse IgG1, κ	SLAMF6	114836	BioLegend	NT-7	
CD354	Mouse IgG1, κ	TREM1	54210	BioLegend	TREM-26	
CD355	Mouse IgG2a, κ	CRTAM	56253	BioLegend	Cr24.1	
CD357	Mouse IgG1, κ	TNFRSF18	8784	BioLegend	621	
CD358/DR6	Mouse IgG1	TNFRSF21	27242	Abcam	DR-6-04-EC	
CD36	Mouse IgM, k	CD36	948	BD Biosciences	CB38 (NL07)	
CD360 (BD)	Mouse IgG1, κ	IL21R	50615	BD Biosciences	17A12	
CD360 (BL)	Mouse IgG1, κ	IL21R	50615	BioLegend	2G1-K12	
CD362	Rat IgG2b	SDC2	6383	R&D Systems	305515	
CD363	Mouse IgG2b	S1PR1	1901	R&D Systems	218713	
CD37	Mouse IgG1, κ	CD37	951	BD Biosciences	M-B371	
CD38	Mouse IgG1, κ	CD38	952	BD Biosciences	HIT2	
CD4	Mouse IgG1, κ	CD4	920	BD Biosciences	RPA-T4	
CD40	Mouse IgG1, κ	CD40	958	BD Biosciences	5C3	
CD41a	Mouse IgG1, κ	ITGA2B/ITGB3		BD Biosciences	HIP8	Recognizes CD41/CD61 complex
CD41b	Mouse IgG3, κ	ITGA2B	3674	BD Biosciences	HIP2	
CD42a	Mouse IgG1, κ	GP9	2815	BD Biosciences	ALMA.16	
CD42b	Mouse IgG1, κ	GP1BA	2811	BD Biosciences	HIP1	
CD43	Mouse IgG1, κ	SPN	6693	BD Biosciences	1G10	
CD44	Mouse IgG2b, k	CD44	960	BD Biosciences	G44-26	
CD45RA	Mouse IgG2b, k	PTPRC	5788	BD Biosciences	HI100	
CD45RB	Mouse IgG1, κ	PTPRC	5788	BD Biosciences	MT4	
CD45RO	Mouse IgG2a, κ	PTPRC	5788	BD Biosciences	UCHL1	
CD46	Mouse IgG2a, κ	CD46	4179	BD Biosciences	E4.3	
CD47	Mouse IgG1, κ	CD47	961	BD Biosciences	B6H12	

Supplementary Table 1. Antibodies included in HT-FC panel, continued.

Antigen	Isotype	NCBI Gene Name	Entrez Gene ID	Supplier	Clone	Note
CD48	Mouse IgM, k	CD48	962	BD Biosciences	TÜ145	
CD49a	Mouse IgG1, κ	ITGA1	3672	BD Biosciences	SR84	
CD49b	Mouse IgG2a, κ	ITGA2	3673	BD Biosciences	12F1	
CD49c	Mouse IgG1, κ	ITGA3	3675	BD Biosciences	C3 II.1	
CD49d	Mouse IgG1, κ	ITGA4	3676	BD Biosciences	9F10	
CD49e	Mouse IgG1, κ	ITGA5	3678	BD Biosciences	IIA1	
CD49f	Rat IgG2a, κ	ITGA6	3655	BD Biosciences	GoH3	
CD5	Mouse IgG1, κ	CD5	921	BD Biosciences	UCHT2	
CD50	Mouse IgG2b, k	ICAM3	3385	BD Biosciences	TÜ41	
CD51/CD61	Mouse IgG1, κ	ITGAV	3685	BD Biosciences	23C6	
CD52	Mouse IgG2b, k	CD52	1043	BioLegend	HI186	
CD53	Mouse IgG1, κ	CD53	963	BD Biosciences	HI29	
CD54	Mouse IgG1, κ	ICAM1	3383	BD Biosciences	HA58	
CD55	Mouse IgG2a, κ	CD55	1604	BD Biosciences	IA10	
CD56	Mouse IgG1, κ	NCAM1	4684	BD Biosciences	B159	
CD57	Mouse IgM, k	B3GAT1	27087	BD Biosciences	HNK-1	
CD58	Mouse IgG2a, κ	CD58	965	BD Biosciences	1C3	
CD59	Mouse IgG2a, κ	CD59	966	BD Biosciences	p282 (H19)	
CD6	Mouse IgG1, κ	CD6	923	BD Biosciences	M-T605	
CD60b	Mouse IgM	N/A	N/A	Lifespan Bioscienc	not provided	9-0-acetylated disialosyl group linked to glycoprotein
CD61	Mouse IgG1, κ	ITGB3	3690	BD Biosciences	VI-PL2	
CD62E	Mouse IgG1, κ	SELE	6401	BD Biosciences	68-5H11	
CD62L	Mouse IgG2a, κ	SELL	6402	BD Biosciences	Sk11	
CD62P	Mouse IgG1, κ	SELP	6403	BD Biosciences	AK-1	
CD63	Mouse IgG1, κ	CD63	967	BD Biosciences	H5C6	
CD64	Mouse IgG1, κ	FCGR1A	2209	BD Biosciences	10.1	
CD65	Mouse IgM	N/A	N/A	Beckman Coulter	88H7	Type II chain fucoganglioside
CD65s	Mouse IgM	N/A	N/A	Abcam	VIM-2	Sialylated form of CD65
CD66	Mouse IgG2a, κ	CEACAM1	109770	BD Biosciences	B1.1/CD66	
CD66b	Mouse IgM, k	CEACAM8	1088	BD Biosciences	G10F5	
CD66c	Mouse IgG1, κ	CEACAM6	4680	BD Biosciences	B6.2/CD66c	
CD66d	Mouse IgG1	CEACAM3	1084	Abcam	CLB-gran/10, IH4Fc	
CD66e	Mouse IgG1	CEACAM5	1048	R&D Systems	487618	
CD69	Mouse IgG1, κ	CD69	969	BD Biosciences	FN50	
CD7	Mouse IgG1, κ	CD7	924	BD Biosciences	M-T701	
CD70	Mouse IgG3, κ	CD70	970	BD Biosciences	Ki-24	
CD71	Mouse IgG2a, κ	TFRC	7037	BD Biosciences	M-A712	
CD72	Mouse IgG2b, k	CD72	971	BD Biosciences	J4-118	
CD73	Mouse IgG1, κ	NT5E	4907	BD Biosciences	AD2	
CD74	Mouse IgG2a, κ	CD74	972	BD Biosciences	M-B741	
CD75	Mouse IgM, k	N/A	N/A	BD Biosciences	LN1	Carbohydrate antigen
CD77	Mouse IgM, k	N/A	N/A	BD Biosciences	5B5	Globotriaosylceramide
CD79a	Mouse IgG1, κ	CD79A	973	R&D	706931	
CD79b	Mouse IgG1, κ	CD79B	974	BD Biosciences	CB3-1	
CD8	Mouse IgG1, κ	CD8A	925	BD Biosciences	HIT8a	
CD80	Mouse IgG1, κ	CD80	941	BD Biosciences	L307.4	
CD81	Mouse IgG1, κ	CD81	975	BD Biosciences	JS-81	
CD82	Mouse IgG2a, κ	CD82	3732	BioLegend	ASL-24	
CD83	Mouse IgG1, κ	CD83	9308	BD Biosciences	HB15e	
CD84	Mouse IgG1	CD84	8832	Biolegend	CD84.1.21	
CD85A	Mouse IgG1	LILRB3	11025	Biolegend	MKT5.1	
CD85D	Rat IgG2a	LILRB2	10288	Biolegend	42D1	
CD85G	Mouse IgG1	LILRA4	23547	BioLegend	17G10.2	
CD85H	Rat IgG2a, κ	LILRA2	11027	Biolegend	24	
CD85J	Mouse IgG2b, k	LILRB1	10859	BD Biosciences	GHI/75	
CD86	Mouse IgG1, κ	CD86	942	BD Biosciences	2331 (FUN-1)	
CD87	Mouse IgG1, κ	PLAUR	5329	BD Biosciences	VIM5	
CD88	Rabbit IgG	C5AR1	728	BD Biosciences	C85-4124	
CD89	Mouse IgG1, κ	FCAR	2204	BD Biosciences	A59	
CD8b	Mouse IgG2a, κ	CD8B	926	BD Biosciences	2ST8.5H7	
CD9	Mouse IgG1, κ	CD9	928	BD Biosciences	M-L13	
CD90	Mouse IgG1, κ	THY1	7070	BD Biosciences	5E10	
CD91	Mouse IgG1, κ	LRP1	4035	BD Biosciences	A2MR-α2	
CD92	Mouse IgG2b	SLC44A1	23446	Abcam	VIM-15b	
CD94	Mouse IgG1, κ	KLRD1	3824	BD Biosciences	HP-3D9	

Supplementary Table 1. Antibodies included in HT-FC panel, continued.

Antigen	Isotype	NCBI Gene Name	Entrez Gene ID	Supplier	Clone	Note
CD95	Mouse IgG1, κ	FAS	355	e-Bioscience	DX2	
CD96	Mouse IgG1	CD96	10225	Biolegend	NK92.39	
CD97	Mouse IgG1, κ	CD97	976	BD Biosciences	VIM3b	
CD98	Mouse IgG1, κ	SLC3A2	6520	BD Biosciences	UM7F8	
CD99	Mouse IgG2a, κ	CD99	4267	BD Biosciences	TÜ12	
CDH3	Mouse IgG1	CDH3	1001	R&D Systems	104805	
CDH6	Mouse IgG1	CDH6	1004	R&D Systems	427909	
CDw198	Rat IgG2b	CCR8	1237	R&D Systems	191704	
CDw199	Mouse IgG2a, κ	CCR9	10803	BD Biosciences	112509	
CDw210	Rat IgG2a, κ	IL10RA	3587	BD Biosciences	3F9	
CDw218a	Mouse IgG1, κ	IL18R1	8809	e-Bioscience	H44	
CDw329	Mouse IgG1, κ	SIGLEC9	27180	BD Biosciences	E10-286	
CDw93	Mouse IgG2b, k	CD93	22918	BD Biosciences	R139	
CLA	Rat IgM, κ	SELPLG	6404	BD Biosciences	HECA-452	Modified form of CD162
CLIP	Mouse IgG1, κ	N/A	N/A	BD Biosciences	CerCLIP	MHC Class II associated invariant chain peptides
DCIR	Mouse IgG1, κ	CLEC4A	50856	BD Biosciences	I3-612	
EGF-R	Mouse IgG2b, k	EGFR	1956	BD Biosciences	EGFR1	
FMC7	Mouse IgM, k	MS4A1	931	BD Biosciences	FMC7	
fMLP-R	Mouse IgG1, κ	FPR1	2357	BD Biosciences	5F1	
HPRC	Mouse IgG1, κ	N/A	N/A	BD Biosciences	BB9	Unknown suface glycoprotein
HLA-A2	Mouse IgG2b, k	HLA-A	3105	BD Biosciences	BB7.2	
HLA-ABC	Mouse IgG1, κ	ABCA1	19	BD Biosciences	DX17	
HLA-DM	Mouse IgG1, κ	HFE	3077	BD Biosciences	MaP.DM1	
HLA-DR	Mouse IgG2b, k	HFE	3077	BD Biosciences	TU36	
ITGB7	Rat IgG2a, κ	ITGB7	3695	BD Biosciences	FIB504	
LTBR	Mouse IgG1, κ	LTBR	4055	BD Biosciences	hTNFR-RP-M12	
MIC A/B	Mouse IgG2a, κ	MICA/MICB	100507436/4277	BD Biosciences	6D4	
PAC-1	Mouse IgM, k	DUSP2	1844	BD Biosciences	PAC-1	
Podoplanin	Rat IgG2a, λ	PDPN	10630	BioLegend	NC-08	
SSEA-3	Rat IgM	FUT4		BD Biosciences	MC631	
SSEA-4	Mouse IgG3	FUT4		BD Biosciences	MC813-70	
Stro-1	Mouse IgM, λ	N/A	N/A	BioLegend	STRO-1	Unknown surface antigen
TCR αβ	Mouse IgM, k	TRA/TRB	6955/6957	BD Biosciences	T10B9.1A-31	
TCR γδ	Mouse IgG1, κ	TRG/TRD	6965/6964	BD Biosciences	B1	
TPBG	Mouse IgG1	TPBG	7162	R&D Systems	524744	
Vβ8 TCR	Mouse IgG2b, k	N/A	N/A	BD Biosciences	JR2	Variable β8 subunit (Vβ8) of α/β T cell receptor (TCR)
Vδ2 TCR	Mouse IgG1, κ	N/A	N/A	BD Biosciences	B6	Variable δ2 subunit (Vδ2) of γ/δ T cell receptor (TCR)
β2-microglobulin	Mouse IgM, k	B2M	567	BD Biosciences	TÜ99	
