# Supplementary material for: Cell Surface Profiling Using High-Throughput Flow Cytometry: A Platform for Biomarker Discovery and Analysis of Cellular Heterogeneity
Source: PLoS One. 2014 Aug 29;9(8):e105602. doi: 10.1371/journal.pone.0105602 (PMC4149490; doi:10.1371/journal.pone.0105602)
Supplement: Table S2 — Validation of antigens influenced by enzymatic digestion. (PDF) [file pone.0105602.s007.pdf]

Table S2. Validation of antigens influenced by enzymatic digestion.

	Control		Trypsin Treated				Collagenase Treated				Dispase Treated				Hyaluronidase Treated				DNAse Treated		
Antigen	%+ Cells	Antigen	%+ Cells	Change in %+	Fold-change	Antige n	%+ Cells	Change in %+	Fold-change	Antigen	%+ Cells	Change in %	Fold-change	Antigen	%+ Cells	Change in %+	Fold-change	Antigen	%+ Cells	Change in %+	Fold-change
BLTA	18.6	BLTA	10.2	-8.4	0.5	BLTA	14.1	-4.5	0.8	BLTA	19.5	0.9	1	BLTA	19.8	1.2	1.1	BLTA	19.6	1	1.1
CD1d	39.4	CD1d	31.8	-7.6	0.8	CD1d	16.7	-22.7	0.4	CD1d	29.1	-10.3	0.7	CD1d	34.6	-4.8	0.9	CD1d	34.2	-5.2	0.9
CD4	27	CD4	18	-9	0.7	CD4	18.9	-8.1	0.7	CD4	17.9	-9.1	0.7	CD4	24.2	-2.8	0.9	CD4	22.4	-4.6	0.8
CD8	0.3	CD8	0.3	-0.07	0.8	CD8	0.7	0.36	2.1	CD8	0.4	0.04	1.1	CD8	0.4	0.01	1	CD8	0.3	-0.05	0.9
CD11b	4.2	CD11b	1.4	-2.83	0.3	CD11b	1.5	-2.74	0.3	CD11b	2.8	-1.39	0.7	CD11b	3.9	-0.31	0.9	CD11b	4	-0.25	0.9
CD20	1.7	CD20	0.5	-1.17	0.3	CD20	0.9	-0.79	0.5	CD20	2.4	0.73	1.4	CD20	0.9	-0.74	0.6	CD20	0.9	-0.78	0.5
CD21	33.3	CD21	17	-16.3	0.5	CD21	21.7	-11.6	0.7	CD21	23	-10.3	0.7	CD21	30.6	-2.7	0.9	CD21	28.4	-4.9	0.9
CD23	22.8	CD23	3.8	-18.98	0.2	CD23	5.9	-16.91	0.3	CD23	12.3	-10.5	0.5	CD23	23.5	0.7	1	CD23	19.9	-2.9	0.9
CD25	4	CD25	1.6	-2.44	0.4	CD25	2.5	-1.45	0.6	CD25	1.7	-2.34	0.4	CD25	3.3	-0.68	0.8	CD25	2.9	-1.08	0.7
CD27	43.1	CD27	18.3	-24.8	0.4	CD27	29.7	-13.4	0.7	CD27	32.5	-10.6	0.8	CD27	41.1	-2	1	CD27	40.7	-2.4	0.9
CD30	44.2	CD30	20.7	-23.5	0.5	CD30	29.8	-14.4	0.7	CD30	26.6	-17.6	0.6	CD30	39.5	-4.7	0.9	CD30	36.9	-7.3	0.8
CD35	7.9	CD35	3.8	-4.04	0.5	CD35	6.3	-1.6	0.8	CD35	4.1	-3.73	0.5	CD35	7	-0.86	0.9	CD35	6.2	-1.69	0.8
CD36	3.7	CD36	2.2	-1.45	0.6	CD36	2	-1.63	0.6	CD36	4.2	0.54	1.1	CD36	2.5	-1.12	0.7	CD36	2.8	-0.83	0.8
CD40	4.1	CD40	1.8	-2.23	0.5	CD40	2.8	-1.25	0.7	CD40	2.4	-1.71	0.6	CD40	3	-1.04	0.7	CD40	2.4	-1.72	0.6
CD41a	5.6	CD41a	2.1	-3.53	0.4	CD41a	1.1	-4.53	0.2	CD41a	3.6	-2.01	0.6	CD41a	1.3	-4.29	0.2	CD41a	1.7	-3.93	0.3
CD42a	7.2	CD42a	1.9	-5.29	0.3	CD42a	1.4	-5.79	0.2	CD42a	3.8	-3.4	0.5	CD42a	2.6	-4.6	0.4	CD42a	7.5	0.29	1
CD42b	1	CD42b	0.1	-0.91	0.1	CD42b	0.2	-0.89	0.1	CD42b	0.2	-0.81	0.2	CD42b	0.3	-0.74	0.3	CD42b	0.5	-0.51	0.5
CD48	55.1	CD48	38.8	-16.3	0.7	CD48	37.4	-17.7	0.7	CD48	44.6	-10.5	0.8	CD48	53.4	-1.7	1	CD48	52.6	-2.5	1
CD51	12	CD51	10.5	-1.5	0.9	CD51	9.4	-2.61	0.8	CD51	9.3	-2.69	0.8	CD51	9.9	-2.08	0.8	CD51	8.5	-3.55	0.7
CD62L	40.8	CD62L	12.9	-27.9	0.3	CD62L	29.8	-11	0.7	CD62L	30.7	-10.1	0.8	CD62L	36.4	-4.4	0.9	CD62L	33.5	-7.3	0.8
CD62P	1.6	CD62P	0.4	-1.27	0.2	CD62P	0.3	-1.3	0.2	CD62P	2.1	0.45	1.3	CD62P	0.8	-0.84	0.5	CD62P	1.3	-0.34	0.8
CD69	6.6	CD69	2.4	-4.16	0.4	CD69	3.3	-3.32	0.5	CD69	2.8	-3.77	0.4	CD69	3.9	-2.74	0.6	CD69	4	-2.63	0.6
CD81	79.3	CD81	75	-4.3	0.9	CD81	68.5	-10.8	0.9	CD81	69.5	-9.8	0.9	CD81	72.1	-7.2	0.9	CD81	65.7	-13.6	0.8
CD87	1.2	CD87	0.1	-1.167	0.1	CD87	0.7	-0.52	0.6	CD87	0.1	-1.133	0.1	CD87	2	0.8	1.7	CD87	2.3	1.02	1.8
CD99	19.7	CD99	18.8	-0.9	1	CD99	7.3	-12.44	0.4	CD99	13.6	-6.1	0.7	CD99	13.1	-6.6	0.7	CD99	20.6	0.9	1
CD106	1.1	CD106	0.7	-0.45	0.6	CD106	0.4	-0.72	0.4	CD106	0.3	-0.83	0.3	CD106	0.4	-0.71	0.4	CD106	0.8	-0.34	0.7
CD108	29.7	CD108	20.3	-9.4	0.7	CD108	17.3	-12.4	0.6	CD108	18.3	-11.4	0.6	CD108	30.2	0.5	1	CD108	27.6	-2.1	0.9
CD109	1.6	CD109	0.9	-0.69	0.6	CD109	1.1	-0.52	0.7	CD109	1.9	0.31	1.2	CD109	2.2	0.61	1.4	CD109	2.1	0.55	1.3
CD118	1.3	CD118	0.9	-0.44	0.7	CD118	0.8	-0.57	0.6	CD118	1.3	-0.05	1	CD118	1.5	0.18	1.1	CD118	1.7	0.32	1.2
CD120A	12.6	CD120A	10.2	-2.4	0.8	CD120A	8.4	-4.17	0.7	CD120A	11.8	-0.8	0.9	CD120A	13.7	1.1	1.1	CD120A	9.6	-2.96	0.8
CD120b	16.3	CD120b	9.8	-6.52	0.6	CD120b	12.4	-3.9	0.8	CD120b	4.8	-11.53	0.3	CD120b	14.4	-1.9	0.9	CD120b	14	-2.3	0.9
CD126	29.4	CD126	18.6	-10.8	0.6	CD126	21.7	-7.7	0.7	CD126	21.8	-7.6	0.7	CD126	25.9	-3.5	0.9	CD126	26.8	-2.6	0.9
CD134	6.3	CD134	3.6	-2.7	0.6	CD134	0.8	-5.46	0.1	CD134	4.1	-2.23	0.6	CD134	7.3	1.01	1.2	CD134	7.3	1.01	1.2
CD138	6.3	CD138	3.7	-2.62	0.6	CD138	1.1	-5.14	0.2	CD138	4.7	-1.61	0.7	CD138	6.3	0.05	1	CD138	6.7	0.39	1.1
CD143	5.3	CD143	0.6	-4.66	0.1	CD143	0.5	-4.83	0.1	CD143	0.6	-4.74	0.1	CD143	0.3	-5.03	0	CD143	0.6	-4.73	0.1
CD146	44.6	CD146	38.8	-5.8	0.9	CD146	40.1	-4.5	0.9	CD146	41.8	-2.8	0.9	CD146	44.6	0	1	CD146	43.5	-1.1	1
CD150	8.5	CD150	4.9	-3.58	0.6	CD150	6.6	-1.86	0.8	CD150	3.6	-4.88	0.4	CD150	6.2	-2.21	0.7	CD150	5.5	-2.91	0.7
CD154	24.1	CD154	10.6	-13.5	0.4	CD154	17.1	-7	0.7	CD154	11.4	-12.7	0.5	CD154	20.6	-3.5	0.9	CD154	20.7	-3.4	0.9
CD157	4.6	CD157	4.3	-0.27	0.9	CD157	5.5	0.88	1.2	CD157	4	-0.56	0.9	CD157	3.3	-1.24	0.7	CD157	3.9	-0.68	0.9
CD162	86.5	CD162	64.9	-21.6	0.8	CD162	68.5	-18	0.8	CD162	70	-16.5	0.8	CD162	86.3	-0.2	1	CD162	86	-0.5	1
CD167	6.1	CD167	6.2	0.08	1	CD167	6.8	0.67	1.1	CD167	5.1	-1.03	0.8	CD167	6.7	0.62	1.1	CD167	7.4	1.25	1.2
CD171	13.4	CD171	26.9	13.5	2	CD171	21.9	8.5	1.6	CD171	18.4	5	1.4	CD171	12.7	-0.7	0.9	CD171	12.9	-0.5	1
CD183	25	CD183	14.1	-10.9	0.6	CD183	14.5	-10.5	0.6	CD183	15.7	-9.3	0.6	CD183	18.2	-6.8	0.7	CD183	15.8	-9.2	0.6
CD191	11.6	CD191	8.8	-2.82	0.8	CD191	5.7	-5.91	0.5	CD191	12	0.4	1	CD191	9.4	-2.23	0.8	CD191	7.7	-3.88	0.7

Supplementary Table 2. Validation of antigens influenced by enzymatic digestion, continued.

	Control		Trypsin Treated				Collagenase Treated				Dispase Treated				Hyaluronidase Treated				DNAse Treated		
Antigen	%+ Cells	Antigen	%+ Cells	Change in %+	Fold-change	Antigen	%+ Cells	Change in %+	Fold-change	Antigen	%+ Cells	Change in %	Fold-change	Antigen	%+ Cells	Change in %+	Fold-change	Antigen	%+ Cells	Change in %+	Fold-change
CD194	0.1	CD194	0	-0.059	0.4	CD194	0	-0.056	0.4	CD194	0.1	0.025	1.3	CD194	0	-0.049	0.5	CD194	0.3	0.165	2.7
CD195	8.6	CD195	3.9	-4.71	0.5	CD195	6.5	-2.16	0.7	CD195	6.8	-1.83	0.8	CD195	8.1	-0.5	0.9	CD195	9	0.41	1
CD196	0.8	CD196	0.3	-0.52	0.3	CD196	0.9	0.14	1.2	CD196	0.6	-0.19	0.8	CD196	1.1	0.26	1.3	CD196	1	0.19	1.2
CD205	36.3	CD205	33.7	-2.6	0.9	CD205	33.1	-3.2	0.9	CD205	29.8	-6.5	0.8	CD205	34	-2.3	0.9	CD205	39.2	2.9	1.1
CD213a2	1.1	CD213a2	0.5	-0.59	0.4	CD213a2	0.3	-0.75	0.3	CD213a2	0.6	-0.49	0.5	CD213a2	0.9	-0.11	0.9	CD213a2	0.9	-0.13	0.9
CD218b	8.4	CD218b	2.7	-5.71	0.3	CD218b	3.9	-4.54	0.5	CD218b	6.4	-2.04	0.8	CD218b	10.8	2.38	1.3	CD218b	11.4	2.98	1.4
CD223	5.4	CD223	10.8	5.43	2	CD223	8.3	2.93	1.5	CD223	5.9	0.51	1.1	CD223	4.1	-1.23	0.8	CD223	5.1	-0.31	0.9
CD229	56.2	CD229	38.2	-18	0.7	CD229	39.8	-16.4	0.7	CD229	45.6	-10.6	0.8	CD229	52.4	-3.8	0.9	CD229	49.7	-6.5	0.9
CD245	88.2	CD245	25.4	-62.8	0.3	CD245	9.2	-79.01	0.1	CD245	86.3	-1.9	1	CD245	63.9	-24.3	0.7	CD245	59.6	-28.6	0.7
CD247	4.8	CD247	0.9	-3.85	0.2	CD247	0.7	-4.04	0.2	CD247	2.1	-2.64	0.4	CD247	3.2	-1.6	0.7	CD247	5.2	0.46	1.1
CD256	0.9	CD256	0.4	-0.51	0.4	CD256	0.5	-0.35	0.6	CD256	1.8	0.92	2.1	CD256	0.6	-0.26	0.7	CD256	0.6	-0.26	0.7
CD257	48.6	CD257	21.3	-27.3	0.4	CD257	18.3	-30.3	0.4	CD257	22.9	-25.7	0.5	CD257	42.7	-5.9	0.9	CD257	43.8	-4.8	0.9
CD258	0.4	CD258	0.7	0.3	1.8	CD258	0.7	0.29	1.8	CD258	0.6	0.22	1.6	CD258	0.7	0.37	2	CD258	0.9	0.54	2.5
CD262	12.8	CD262	22.7	9.9	1.8	CD262	19.3	6.5	1.5	CD262	16.8	4	1.3	CD262	13.4	0.6	1	CD262	12.1	-0.7	0.9
CD267	14.9	CD267	8.8	-6.12	0.6	CD267	11.3	-3.6	0.8	CD267	9.9	-4.96	0.7	CD267	9.2	-5.66	0.6	CD267	9.1	-5.79	0.6
CD269	6.4	CD269	10.2	3.85	1.6	CD269	3	-3.35	0.5	CD269	8.8	2.42	1.4	CD269	5.9	-0.44	0.9	CD269	2.6	-3.74	0.4
CD277	1.3	CD277	1.6	0.3	1.2	CD277	1.7	0.46	1.4	CD277	1.2	-0.04	1	CD277	1.7	0.45	1.4	CD277	1.8	0.49	1.4
CD288	1.6	CD288	0.8	-0.8	0.5	CD288	8	6.39	5.1	CD288	5.3	3.78	3.4	CD288	0.6	-0.97	0.4	CD288	5.1	3.53	3.3
CD300d	4.2	CD300d	1.2	-3.03	0.3	CD300d	1.7	-2.55	0.4	CD300d	1.6	-2.59	0.4	CD300d	6.1	1.88	1.4	CD300d	5.9	1.62	1.4
CD300e	2.4	CD300e	0.7	-1.61	0.3	CD300e	1.1	-1.22	0.5	CD300e	0.5	-1.85	0.2	CD300e	1.1	-1.22	0.5	CD300e	3.4	1.03	1.4
CD304	13	CD304	4.5	-8.51	0.3	CD304	10.4	-2.6	0.8	CD304	19.7	6.7	1.5	CD304	17	4	1.3	CD304	17.6	4.6	1.4
CD307	1.6	CD307	0.9	-0.73	0.5	CD307	0.7	-0.93	0.4	CD307	0.9	-0.69	0.6	CD307	1.4	-0.19	0.9	CD307	1.2	-0.43	0.7
CD338	1.8	CD338	16.9	15.08	9.3	CD338	7.1	5.25	3.9	CD338	13.2	11.38	7.3	CD338	9.4	7.56	5.2	CD338	7.6	5.74	4.2
GMA DTA	9.5	GMA DTA	8.3	-1.22	0.9	GMA DTA	10	0.46	1	GMA DTA	5.5	-3.97	0.6	GMA DTA	11.5	1.99	1.2	GMA DTA	8.1	-1.4	0.9
HLADM	0.3	HLADM	0.4	0.12	1.4	HLADM	0.4	0.14	1.5	HLADM	0.3	0.03	1.1	HLADM	0.4	0.11	1.4	HLADM	0.4	0.16	1.6
ITGB7	38.6	ITGB7	20	-18.6	0.5	ITGB7	19.2	-19.4	0.5	ITGB7	28.8	-9.8	0.7	ITGB7	32.6	-6	0.8	ITGB7	30.2	-8.4	0.8
KIR-NKAT2	0.2	KIR-NKAT2	0.3	0.07	1.3	KIR-NKAT2	0.2	0	1	KIR-NKAT2	0.3	0.04	1.2	KIR-NKAT2	0.1	-0.1	0.6	KIR-NKAT2	0.4	0.2	1.9
MIC A&B	24.6	MIC A&B	29.6	5	1.2	MIC A&B	29.4	4.8	1.2	MIC A&B	33.1	8.5	1.3	MIC A&B	28.8	4.2	1.2	MIC A&B	27.2	2.6	1.1
TWEAK	0.1	TWEAK	1.3	1.24	13.4	TWEAK	1.9	1.75	18.5	TWEAK	2.1	1.95	20.5	TWEAK	2.7	2.63	27.3	TWEAK	2.7	2.62	27.2
p-CAD	2.1	p-CAD	0.4	-1.69	0.2	p-CAD	0.2	-1.88	0.1	p-CAD	1.1	-0.96	0.5	p-CAD	1.1	-0.94	0.5	p-CAD	2.1	0.07	1
CD100	46.5	CD100	28.6	-17.9	0.6	CD100	38.7	-7.8	0.8	CD100	31.2	-15.3	0.7	CD100	49.5	3	1.1	CD100	45	-1.5	1
CD175s	29.7	CD175s	34.9	5.2	1.2	CD175s	42.6	12.9	1.4	CD175s	24.1	-5.6	0.8	CD175s	52.3	22.6	1.8	CD175s	31.2	1.5	1.1
CD227	13.6	CD227	13.5	-0.1	1	CD227	12.9	-0.7	0.9	CD227	13	-0.6	1	CD227	15.7	2.1	1.2	CD227	15.3	1.7	1.1
PAC-1	0.1	PAC-1	0.1	-0.02	0.8	PAC-1	0.1	-0.01	0.9	PAC-1	0.2	0.04	1.3	PAC-1	0.1	-0.078	0.4	PAC-1	0.1	-0.02	0.8
CD349	1.6	CD349	4.1	2.54	2.6	CD349	1.3	-0.32	0.8	CD349	2.2	0.64	1.4	CD349	2.1	0.48	1.3	CD349	2.4	0.75	1.5
Number affected:		13		30	36	12		29	30	4		25	17	2		10	12	1		10	8

Antigens highlighted in yellow showed both 2-fold relative and 5% absolute difference in at least one enzyme condition, and are listed separately in Supplementary Table 3.

Red and green indicates decreased and increased expression respectively. Changes of ≥5% or ≥2x are highlighted. N, normal control; H hyaluronidase; C, collagenase; D, dispase; T, trypsin; DN, DNase.
