# Supplementary material for: Cell Surface Profiling Using High-Throughput Flow Cytometry: A Platform for Biomarker Discovery and Analysis of Cellular Heterogeneity
Source: PLoS One. 2014 Aug 29;9(8):e105602. doi: 10.1371/journal.pone.0105602 (PMC4149490; doi:10.1371/journal.pone.0105602)
Supplement: Table S3 — The 22 most-affected antigens appear predisposed to altered detection by enzymatic digestion. (PDF) [file pone.0105602.s008.pdf]

Table S3. The 22 most-affected antigens appear predisposed to altered detection by enzymatic digestion. Several antigens have a soluble as well as membrane-bound form, some have a known extracellular cleavage site, and others are biologically active upon release from the cell surface.

	Zola, Swart, Nicholson, Voss	Other literature
**CD1d**	---	"In the late endosomes, the CD1d complex is modified, such that after trafficking back to the plasma membrane..." http://www.nature.com/nri/journal/v3/n1/abs/nri979.html
**CD23**	Continuous proteolytic cleavage of membrane-bound CD23 results in soluble CD23 molecules of varying size	Also exists as a soluble excreted form. http://www.uniprot.org/uniprot/P06734
**CD27**	Soluble form is released by activated lymphocytes	"After activation of lymphocytes, a soluble form of CD27 is released into the extracellular environment, most likely via proteolytic cleavage" http://iai.asm.org/cgi/reprint/68/5/3036.pdf
**CD30**	Soluble form of CD30 is released by metalloproteinase	"Finally, transmembrane signalling by CD30 might be regulated by a soluble form of CD30 that is released by proteolytic cleavage of membrane-anchored CD30 http://www.sciencedirect.com/science/article/pii/S1471489204000852
**CD42a**	---	Hale et al. (2002) showed that in vitro bromelain treatment of leukocytes in whole blood proteolytically altered 14 of 59 leukocyte markers studied http://www.pjbs.org/pjnonline/fin975.pdf
**CD62L**	There is 15 amino acid spacer between the membrane proximal CCP domain and the transmembrane region, which contains the proteolytic cleavage site (Lys283-Ser284)	Constitutively expressed bromelain-sensitive molecules included CD7, CD8α, CD14, CD16, CD21, CD41, CD42a, CD44, CD45RA, CD48, CD57, CD62L, CD128a, and CD128b http://www.sciencedirect.com/science/article/pii/S152166160295254X
**CD99**	---	"In this paper we demonstrate that CD99 localized at the endothelial cell junctions is sensitive to proteolysis by gingipains" http://www.ncbi.nlm.nih.gov/pmc/articles/PMC1418641/
**CD120b**	CD120b is readily cleaved by the metalloproteinase TACE into a soluble form that retains the ability to bind TNF	BAL cells express and shed TNF-Rs [CD120a, CD120b], as is known for cells of other body compartments http://www.sciencedirect.com/science/article/pii/S1043466698904690
**CD134**	Member of the TNF/NGF receptor family (see CD27, CD30, CD40, CD95, CD120a, CD120b, CD137).	The transient nature of CD134 and CD137 expression on activated CD4+ T cells is the result of proteolytic shedding http://onlinelibrary.wiley.com/doi/10.1002/1521-4141%28200212%2932:12%3C3617::AID-IMMU3617%3E3.0.CO;2-M/pdf
**CD138**	The extracellular region contains five potential glycosaminoglycan attachment sites and a protease cleavage site at its C-terminus.	"Owing to the sensitivity of CD138 towards proteolytic cleavage" http://www.finels.com/product/up_files/M7228.pdf
**CD143**	Soluble forms of CD143 are detectable in plasma, urine, amniotic and cerebrospinal fluids and seminal plasma	Soluble serum ACE originates from endothelial cells by proteolytic cleavage by an unidentified protease of the Arg^{1203}–Ser^{1204} peptide bond in the stalk region near the C-terminal transmembrane sequence of the ACE molecule. www.clinchem.org/cgi/content/full/51/6/1040
**CD154**	Soluble forms of CD154 are produced as a result of intracellular cleavage by a matrix metalloproteinase in activated cells.	Furthermore, soluble CD154 generated through proteolytic cleavage of the extracellular domain is biologically active, especially in multimeric forms. http://www.ncbi.nlm.nih.gov/pmc/articles/PMC1539120/
**CD171**	The CD171 ectodomain can be shed as a result of post-translational cleavage by disintegrin metalloproteinase (ADAM). Plasmin can also cleave	Shed as large ectodomain fragments as a result of posttranslational cleavage http://prow.nci.nih.gov/guide/1813332452_g.htm

	CD171 at the same site.	
**CD191**	---	---
**CD218b**	---	---
**CD223**	---	A soluble form of human CD223 has been detected by ELISA in human serum http://www.jimmunol.org/content/173/11/6806.long
**CD245**	---	The expression level of CD245 on lymphocytes can be upregulated after 3-7 days stimulation with PHA. It has been reported that CD245 is involved in signal transduction and costimulation of T and NK cells. www.biolegend.com/pe-anti-human-**cd245-p220-240**-5039.html
**CD257**	The extracellular domain is shed as a result of proteolysis in the extracellular stalk region.	An active, soluble homotrimer form can be released by proteolytic cleavage www.copewithcytokines.org/cope.cgi?key=BLyS
**CD288**	---	BAFF is … expressed as a transmembrane protein on various cell types …. The transmembrane form can be cleaved from the membrane, generating a soluble protein fragment. http://en.wikipedia.org/wiki/B-cell_activating_factor
**CD304**	A soluble isoform (sNRP1) consists of the CUB and Factor V/VIII homology domains only.	---
**CD338**	---	---
**ITGB7**	---	---
**TWEAK**	A soluble isoform of CD255 consisting of 156 amino acids comprising the recptor binding portion of the ectodomain results from proteolytic cleavage by furin.	A soluble form of TWEAK is generated from the membrane-associated molecule by proteolytic cleavage after Arg 93, suggesting that TWEAK may have long-range effects. TWEAK is expressed widely in many tissues and cells. http://www.ncbi.nlm.nih.gov/pmc/articles/PMC2878507/

Most information from "*Leukocyte and Stromal Cell Molecules: The CD Markers*", by Heddy Zola, Bernadette Swart, Ian Nicholson and Elena Voss, Wiley, 2007 (http://wiley.com/WileyCDA/WileyTitle/productCd-0471701327.html) supplemented with other literature and sources as indicated.
