# Supplementary material for: Cell Surface Profiling Using High-Throughput Flow Cytometry: A Platform for Biomarker Discovery and Analysis of Cellular Heterogeneity
Source: PLoS One. 2014 Aug 29;9(8):e105602. doi: 10.1371/journal.pone.0105602 (PMC4149490; doi:10.1371/journal.pone.0105602)
Supplement: Table S4 — Fixation following antibody staining significantly alters detection of a small proportion of markers. (PDF) [file pone.0105602.s009.pdf]

**Table S4. Fixation following antibody staining significantly alters detection of a small proportion of markers.**

| Antigen | Control | Fixation Post-Stain | Absolute Change | Fold Change | Notes and Reference |
|---|---|---|---|---|---|
| CD53 | 11.3 | 3.12 | 8.2 | 0.28 | HI29 induced homotypic cell aggregation of lymphoid cell lines http://www.sciencedirect.com/science/article/pii/S0171298597800587 |
| CD74 | 9.0 | 33.1 | 24.1 | 3.67 | No specific information on this antibody, antigen, epitope |
| CD109 | 7.4 | 2.3 | 5.2 | 0.30 | Some epitopes disrupted by SDS http://bloodjournal.hematologylibrary.org/content/99/5/1692.full.pdf |
| CD114 | 11.5 | 2.9 | 8.6 | 0.25 | Raised against intact cell membrane protein http://www.sciencedirect.com/science/article/pii/S0301472X10003541 |
| CD134 | 14.1 | 6.2 | 7.9 | 0.44 | Undergoes conformational change with eGFP tag http://jvi.asm.org/content/80/7/3386.full.pdf |
| CD159c | 15.5 | 0.48 | 15.0 | 0.03 | No specific information on this antibody, antigen, epitope |
| CD200 | 15.3 | 5.6 | 9.7 | 0.37 | No specific information on this antibody, antigen, epitope |
| CD201 | 14.8 | 4.8 | 10.0 | 0.32 | Antibody blocks activated protein C binding to endothelial protein C receptor http://www.copewithcytokines.de/cope.cgi?key=CD201__REFERENCES |

A number of other epitopes showed > 10% absolute change without reaching a two-fold relative difference, including β2-microglobulin, CD44, CD47, CD92, CD96, CD105, CD111, CD119, CD138, CD166, CD221, CD222, CD223, CD257, CD340, CD344, MICA/B and LTβR.
