# Supplementary material for: Cell Surface Profiling Using High-Throughput Flow Cytometry: A Platform for Biomarker Discovery and Analysis of Cellular Heterogeneity
Source: PLoS One. 2014 Aug 29;9(8):e105602. doi: 10.1371/journal.pone.0105602 (PMC4149490; doi:10.1371/journal.pone.0105602)
Supplement: Table S5 — Antigens with significant alteration in detection after cryopreservation and thawing. (PDF) [file pone.0105602.s010.pdf]

Table S5. **Antigens with significant alteration in detection after cryopreservation and thawing.**

	Control (%)	Cryopreservation (%)	Absolute Change (%)	Fold Change
CD138	30.9	10.5	-20.4	0.34
CD18	17.4	36.3	18.9	2.09
CD186	0.39	5.38	4.99	13.79
CDw199; CCR9	0.55	7.85	7.3	14.27
CD277	0.15	6.94	6.79	46.27
CD284	1.59	17.6	16.01	11.07
CD288	2.63	50.8	48.17	19.32
CD337	7.07	25.8	18.73	3.65
CD300C	0.79	14.9	14.11	18.86
