# Supplementary material for: Cell Surface Profiling Using High-Throughput Flow Cytometry: A Platform for Biomarker Discovery and Analysis of Cellular Heterogeneity
Source: PLoS One. 2014 Aug 29;9(8):e105602. doi: 10.1371/journal.pone.0105602 (PMC4149490; doi:10.1371/journal.pone.0105602)
Supplement: Table S7 — Clusters of antigens expressed on primary ccRCC cancer and stromal cell populations. (PDF) [file pone.0105602.s012.pdf]

Table S7. Clusters of antigens expressed on primary ccRCC cancer and stromal cell populations.

Immune cells cluster

	229 CD45+	243 CD45+	266 CD45+	267 CD45+	278 CD45+	284 CD45+	229 Endothel	243 Endothel	266 Endothel	267 Endothel	278 Endothel	284 Endothel	229 Fibroblas	243 Fibroblas	266 Fibroblas	267 Fibroblas	278 Fibroblas	284 Fibroblas	229 Lin-	243 Lin-	266 Lin-	267 Lin-	278 Lin-	284 Lin-
CD85a	13.8	27.7	35.1	43.2	29.2	36.2	0.253	5.45	24.1	69.1	3.17	5.97	6.09	7.52	6.98	34.2	28.4	8.33	0.308	0.695	11.1	22.4	8.25	3.75
CD32	71.1	38.9	44.4	73.4	46.1	48.4	17.7	41.1	20.8	50.9	20	12.9	66.3	47.9	3.57	67	35.9	21.8	41.4	31.5	15.4	61.9	11.3	9.43
CD86	45.9	25.6	33.3	62.5	23.1	33.7	6.97	5.85	16.1	40.8	2.63	2.71	37.3	5.59	5.83	52.7	5.29	0.394	19.5	2.1	12.4	52.9	1.34	0.288
CD170	49.2	27.3	28.9	59.9	25.3	25.3	7.14	39.4	34.9	52.7	14	3.05	77.1	15.2	4.4	52.8	82.4	9.57	4.12	2.44	14.5	40.4	19.6	3.29
CDW198	42.5	28.4	67.8	65.1	43.6	48.7	7.48	15	57.7	30.7	32	16	63.3	12.5	22.5	53.7	59.9	32.5	17.1	1.69	27.5	39.1	36.1	16.9
CD11B	59.6	28.5	40.3	62.7	45.8	41.6	7.6	3.77	25	24.9	6.67	2.22	50	4.9	9.76	30.9	41.5	4	28.5	0.821	12.4	32.3	27.7	0.915
CD96	18.6	19.1	32.1	42.6	58.2	37.6	6.25	1.45	26.2	47	11.2	8.33	45.9	1.96	11.7	55.3	48.4	29	16.3	0.78	15.9	53.4	17.4	11.3
CD319	47.9	26.7	50.3	77.6	56	65.7	20.1	7.28	28.8	55.6	17.3	21.2	66.5	8.63	8.45	63.4	53.7	47	40.6	3.03	15.2	59.7	26.7	33.2
CD60b	45.2	55.1	64.7	46.1	79.4	63	61.6	17.5	58.5	44.1	62.9	12.5	55.7	17.4	34	36.5	35.7	52.7	44.4	12.3	33.8	24.8	30.7	16.3
CD172g	36.2	18.3	41.9	37.3	75.5	47.6	1.97	5.17	20	19.8	2.78	12.9	1	1.27	2.38	33.3	27.6	26.6	1.39	0.349	8.77	30.3	20.9	19.4
CD195	27.8	43	40.2	54.6	84.8	48.7	1.56	1	33.3	12.3	9.59	3.45	10.4	9.09	33.3	24.1	15.1	3.6	1.79	0.946	14.3	15.9	5.8	2.07
CD45RB	96.1	21.7	54.7	67.6	96.6	88	0.242	12.1	1	0.595	10.3	3.8	7.69	51.5	7.69	37.3	7.26	0.361	4.69	0.615	1	2.37	1.47	0.759
CD53	76.3	51.8	66.8	88.8	91.6	96.8	1.33	11.9	1	14.9	16.9	15.4	31.5	19.4	10	17.2	20	6.16	9.01	5.65	1	8.46	7.99	3.16
CD229	58.1	27.5	57.4	49.2	76.4	69	0.893	1	3.23	10.8	6.41	3.03	4.84	1	1.61	13.4	20.1	11.9	1.45	29.2	4.03	21.6	5.19	6.36
CD4	61	49	35.7	65	67.9	56	0.828	8.62	4.35	3.42	10	2.9	22.8	31.7	7.95	21.1	61.7	15.4	4.52	1.41	4.76	10.3	3.19	0.834
CD305	76.8	44.9	68.5	84.3	44.1	78.2	10.1	14.4	33.8	23.7	5.8	5.46	43	34.2	10.9	43.8	40.8	14.8	7.2	2.59	19.2	24.8	2.71	1.33
CD11A	95.6	94.8	79.3	90.5	97.4	47.1	3.11	14.4	13	33.2	14.8	2.32	36	25.6	12.2	48.5	32.7	3.36	46.3	42.4	11.5	44	41.6	7.21
CD45	95.3	98.8	99.9	97.7	97.5	50.3	1.28	32	1	16	15.1	5.11	13.4	52.5	5.88	33	17.9	3.54	28.2	33.4	9.62	25.3	20.4	5.95
CD28	44.5	35.4	37	32.9	68.8	44.5	4.67	2.5	7.14	6.67	20.3	1.79	37.1	0.877	1	18.3	31.3	1.11	28.2	0.567	1.4	14.1	26	0.423
CD2	100	64.5	50.7	48.4	89.3	52.5	1	4.42	2.17	1.81	9.59	4.06	NaN	3.36	2.59	13	15.7	4.18	25	17.1	7.83	21.8	49.9	8.87
CD6	52.7	39.4	49.8	44.9	79.3	54.5	0.794	1.79	1.9	10.5	5	2.93	8.8	0.735	4.9	22.7	15.8	1.92	43.8	3.28	9.51	22	32.1	1.04
CD52	64.7	50.7	58	56.6	88.1	77	1.55	1.11	3.23	9.59	4.76	1.56	8.9	1.55	0.901	15	18.5	1.6	47.6	0.452	2.46	16.1	18.9	2.05
CD7	37.1	52.5	41.8	36.2	55.3	49.8	4.93	4	3.61	5.88	13.3	0.68	20	2.63	1.09	11.9	35	2.22	8.06	0.412	2.83	8.28	14.6	0.336
CD3	37.1	52.7	49.2	33.4	77.5	50.1	0.288	2.61	1.04	1	3.95	1.73	3.17	0.995	1.09	1.5	6.83	1	9.71	1.09	0.372	0.816	4.58	0.236
CD5	38.3	53	52.5	36.5	74.5	43.4	0.535	1	1	0.935	3.33	4.27	6.52	0.676	1	4.63	13.6	0.984	23.9	1.31	4.22	4.16	9.82	0.908
CD69	46.2	49	36.7	28.4	73.8	42.1	1.24	0.613	5.45	1.34	1.32	4.81	3.48	1.06	1	2.88	10.4	1	4.22	0.351	1.48	1.42	11.3	0.945
CD45RO	72.5	90.7	59.4	82.8	92.4	55.6	1.03	6.25	5	6.29	6.06	2.21	12.2	8.82	5.88	10.4	17.1	2.65	9.25	0.593	2.38	7.09	7.48	2.73
CD18	94.6	96.2	95.4	96.6	99	96.9	1.29	6.54	11.8	19.7	14.7	2.36	20.2	8.77	18.2	29.3	15.2	1.42	11.6	7.08	18.3	31.1	7.79	3.19
CD48	72.3	61.8	72.8	66.6	81.2	85.4	1	1	1	0.639	2.41	4.8	0.995	1.14	1	0.581	4.68	1	1.49	0.198	4.17	28.2	0.912	0.385
CD43	63.1	71.1	83.9	67.9	91.4	65.8	0.22	8	1	1.76	2.3	0.99	3.52	0.885	1	3.08	6.01	1	4.59	0.838	3.51	4.31	4.47	0.62
CD352	45.2	61.6	69.8	46.7	83.5	68.3	1.04	3.77	7.95	1.98	4.65	4.64	3.29	1.48	1	4.57	4.72	1.33	0.885	1.37	4.14	2.61	1.85	1.5
CD50	69.2	77	80.6	66.6	93.7	92	49.9	25	67.6	27	31.9	31.1	1.29	1.71	1	1.97	4.65	1.15	0.612	0.837	4.81	3.11	0.723	0.19
HLA-DR	63.3	37.6	41.6	60.2	47.2	47.7	5.93	42.3	68.9	16.6	47.7	30.8	21.2	26.2	1	16.9	36.2	10	2.65	22.4	3.62	2.57	13.2	5.56

Endothelial cells clusters

	229 CD45+	243 CD45+	266 CD45+	267 CD45+	278 CD45+	284 CD45+	229 Endothel	243 Endothel	266 Endothel	267 Endothel	278 Endothel	284 Endothel	229 Fibroblas	243 Fibroblas	266 Fibroblas	267 Fibroblas	278 Fibroblas	284 Fibroblas	229 Lin-	243 Lin-	266 Lin-	267 Lin-	278 Lin-	284 Lin-
CD73	18.7	10.1	5.45	10.2	23.8	11.6	93.7	93.7	97.1	94	97.4	89.9	53.9	17.3	38.8	68.9	57.1	29.1	80.4	59.2	21.9	81.9	96.9	47.6
CD231	37.8	3.12	29.8	45	17.6	12.6	52.4	88.3	86.2	88.1	70.3	80.8	59.5	25.8	22	64.4	46.6	63.6	53.2	2.4	36.9	53.7	31.2	27.2
CD123	29.6	5.31	20.5	40.9	34.7	21.6	91.2	95.2	100	96.5	80	98.9	46.8	8.12	28.1	53.4	48.9	15.6	24.6	1.8	18.5	49.1	35.8	3.23
CD66	14.1	5.94	7.66	10.7	19.6	6.43	74.9	60.3	95.4	80.3	70.8	70.5	20.1	3.98	2.25	19.2	12.3	1.31	4.72	52.2	1.03	5.84	24.3	0.821
CD66d	32	6.07	9.81	17.9	13.8	15	64	45.6	77.6	70.1	53.4	59.7	49.1	2.86	8.7	15.4	17.9	5.34	31.3	38.4	8.18	11.1	15.4	5.28
CD220	3.2	0.868	5.69	4.03	4.37	3.57	91.8	77.9	97.6	81.5	87.7	77.5	12	1.1	3.92	8.14	27	8.04	3.65	30.5	13.3	4.96	5.67	7.63
CD144	6.13	1.47	0.574	3.43	3.56	2.21	99.8	100	100	99.3	100	98.7	40.4	5.68	8.86	11.7	25.9	13.1	11.1	0.613	2.96	1.82	5.57	2.75
HPC	3.48	2.3	3.27	6.59	8.37	7	89.9	80	92	86	82	95.4	34.2	9.49	3.49	24.1	47.6	32.2	1.31	2.07	1.31	4.77	7.01	8.04
CD77	1.86	6.3	3.96	0.811	0.234	1.37	42.7	9.86	18.8	0.667	44	13.6	41.6	3.95	4.55	1.96	18.8	72.8	7.61	3.28	1.19	1.73	0.855	6.23
CD201	8.3	1.9	0.229	1.82	2.81	2.04	58.4	43.9	14.3	25.8	77.2	40.8	79.6	9.21	2.94	28.8	27.9	47.4	81.1	0.181	0.645	4.67	61.7	6.74
CD202b	48.5	31.9	44.6	69.1	24.1	35.7	97.2	48.7	93.3	69.9	98.6	92.4	76.3	43.4	52.1	68.7	51.9	47.8	56.3	2.83	49.7	64.1	29.5	36.6
CD269	12.6	28.5	13.1	40	9.97	16.9	91.7	6.2	92.6	9.57	94	88.7	59.2	13.5	14.5	20	66.2	41	6.43	0.569	8.79	12.5	5.79	11.3
CD292	23.8	1.95	4.27	5.05	10.1	7.28	74.1	10.6	71.9	37.9	96.6	3.42	58.3	6.74	13.9	13.6	57.1	12.9	20.8	1.28	6.7	6.04	8.13	4.71
CD309	39.4	13.9	16.3	42.9	14.3	5.24	68.5	27.9	58.8	44.2	65.8	32.9	70.4	5.02	5.81	31.2	68.3	17.9	18.8	0.484	17.4	29.7	5.94	5.74
CD332	5.18	0.156	2.21	2.14	6.27	0.677	81	1	22.2	4.98	59.3	41.6	60.2	7.14	5.41	3.36	100	34.3	5.81	0.804	1	0.412	49.3	1.14

Fibroblasts cluster

	229 CD45+	243 CD45+	266 CD45+	267 CD45+	278 CD45+	284 CD45+	229 Endothel	243 Endothel	266 Endothel	267 Endothel	278 Endothel	284 Endothel	229 Fibroblas	243 Fibroblas	266 Fibroblas	267 Fibroblas	278 Fibroblas	284 Fibroblas	229 Lin-	243 Lin-	266 Lin-	267 Lin-	278 Lin-	284 Lin-
CD156b	53	33.6	56.7	72.4	51.3	48.5	55.5	70.7	94.7	66.7	80	75.5	98.3	86.9	46.2	87.9	100	98.1	66.9	55.4	44.5	65.1	77.7	57.9
CD111	4.3	2.75	16.5	14.5	15.1	12.1	10.3	24.5	24.5	26.5	19.3	11.9	94.2	99.1	20.6	78.5	99.5	86.8	16.3	20.3	12.3	28.1	55.9	27.5
CD140B	12.1	2.73	15.9	17.8	19.7	4.72	15.7	22.5	23.4	19.2	21.2	6.61	94.9	97.1	24.6	82.6	98.6	94.1	11	3.17	12.4	25	21.8	5.98
CD271	3.56	2.68	2.48	12.1	2.68	3.54	6.32	34.1	32.4	20.2	8.57	6.38	82.4	93.9	23.5	74.4	79.6	85	3.89	1.85	3.29	17.5	1.54	4.96

Cancer cells cluster

	229 CD45+	243 CD45+	266 CD45+	267 CD45+	278 CD45+	284 CD45+	229 Endothel	243 Endothel	266 Endothel	267 Endothel	278 Endothel	284 Endothel	229 Fibroblas	243 Fibroblas	266 Fibroblas	267 Fibroblas	278 Fibroblas	284 Fibroblas	229 Lin-	243 Lin-	266 Lin-	267 Lin-	278 Lin-	284 Lin-
CD26	43.1	12	57	37.6	64.8	60.2	56.2	65	100	93.7	80	85	85.5	8.62	99.1	90.9	80.2	81.9	95.3	65.3	98.5	96	99.9	88.1
CD10	5.95	5.1	5.73	9.69	43.8	10.4	2.05	20.7	51.5	25.5	41.4	16.9	31.3	2.76	92.4	65.5	79.4	16.9	60.2	91.4	87.6	89.5	99.3	70
CAIX	30.4	4.39	42.9	39.3	57.5	41.8	21.2	6.25	82.5	28	55.4	47.5	74.2	8	92.9	76.1	74.1	73.1	97.7	93	96.6	97.3	99.9	86.8
CD24	52.1	9.11	16.4	23.5	61.6	40.6	40.5	13.7	82.2	58.1	62	43.8	89.3	17.9	89.5	91.5	88.8	55.9	93.9	96.6	98.7	97.2	99.8	90
CD203c	41.4	6.54	41	32.9	46.9	33.5	33.9	10.5	94.3	68.9	37.7	39	88.5	19.5	92.8	89	70.2	58.3	88.6	98.2	98.2	97.9	99.8	88
CD245	15	2.46	38.2	51.3	38.5	25.7	4.5	1	41.9	52.4	23.4	15.2	55.8	9.09	67.9	81.4	58.8	46.8	68.8	88.6	86.9	95.6	70.9	38.7
CLA	10.5	7.67	14.5	11.2	8.4	28	24.9	14.3	91.7	7.25	16.9	31	31.2	13.3	60	36.9	12	55.6	49.4	62.3	55.3	72.4	19.3	54.2
CD213a2	2.19	8.63	2.99	53.9	2.68	1.83	1.64	8.33	1	13.7	7.25	2.65	1.32	1	1	40.6	15.1	1.98	3.03	44.8	9.18	86.1	19.9	2.87
CD172a	87.4	37	51.3	77.9	82.5	31.3	47.1	72.2	62.3	77.4	56.4	4.76	71.1	15.5	87.3	71.3	62.2	14.3	99	89.8	97.6	96.3	99.7	65.1
CD66C	8.36	3.87	24.7	33.3	6.49	3.68	3.08	33.3	45.7	52.2	12.2	7.04	21.1	10.7	24.6	61.1	23	5.92	62	26.1	36.6	54.3	60.9	38
CD243 (BC	17.7	2.39	36.6	47.6	19.8	10.4	4.5	2.9	44.4	40.7	5.06	4.26	26.8	2.33	22.9	50.9	11.8	4.51	55.7	4.28	33.9	51.1	38.8	16.5
CD270	58	16.6	48.1	53.2	56.1	53.5	47.4	21.5	52.5	49.5	19.2	12.7	73.6	8.43	58.1	66.5	34.6	17.8	92.8	22.4	57	74.6	93.1	36.1
CD325	7.32	1.84	16.2	25.3	3.52	4.06	2.31	1.49	25	27.7	7.95	2.46	27.5	1.02	4.8	35.4	15.8	4.68	96.3	0.477	14.7	39.2	98	17.7
CD70	20.3	6.8	5.61	7.65	42.8	28.8	8.03	1.06	6.59	1.01	15.7	14.9	46.8	1.71	28.8	14.4	50.6	25.2	98.8	76.6	39	29.1	99.7	89.1
CD318	36.7	20.3	16	48.1	18.3	18.5	4.74	1.61	5.45	12.2	8	9.09	30.1	1.4	1.32	42.8	28.7	19	72.1	73.6	4.75	54.1	88.3	62
CD326	5.12	4.06	2.18	3.47	2.39	7.79	2.56	3.77	2.86	6.21	3.75	18.4	21.3	3.35	59.8	38.8	10.6	30.7	72.5	80.7	33.6	73.4	62.5	74.3
CD56	9.36	7.2	18.9	26.9	19.6	14.2	2.27	5.81	1	24.9	7.5	4.82	22.8	7.02	2.38	57.5	23.3	6.32	55.4	13.4	47.5	79.4	95	55.6
EGF-R	2.91	1.55	0.727	7.35	1.25	1.69	0.241	3.74	2.86	5.94	5.26	4.85	7.44	4.57	12.8	39.9	10.1	3.8	37.9	59.3	47	90.8	86.4	31.3

Supplementary Table 7. Clusters of antigens expressed on primary ccRCC cancer and stromal cell populations, continued.

Immune cells and fibroblasts cluster

	229 CD45+	243 CD45+	266 CD45+	267 CD45+	278 CD45+	284 CD45+	229 Endothel	243 Endothel	266 Endothel	267 Endothel	278 Endothel	284 Endothel	229 Fibroblas	243 Fibroblas	266 Fibroblas	267 Fibroblas	278 Fibroblas	284 Fibroblas	229 Lin-	243 Lin-	266 Lin-	267 Lin-	278 Lin-	284 Lin-
CD38	37.8	54.7	41.7	47	54.7	58.6	1.36	36.5	21.4	34.2	22.6	19.7	13.8	56.1	31.3	73.2	21.7	13.8	2.88	9.71	5.56	24.3	36.2	2.56
CD49D	83.8	88.2	98.5	96.6	98.3	95.4	10.3	44.2	62.5	24.7	18.2	11.2	93.9	98.4	73.3	80.5	98.2	93.4	4.23	8.02	8.93	26.8	8.49	7.78
CD97	16.5	24.4	47.2	44.6	67	59.3	0.239	3.48	1.14	1.05	5.75	3.48	23.5	21.8	3.09	19.4	37.3	67.3	0.88	0.751	1.6	2.82	1.16	2.7
CD14	48.3	31.8	33.3	62.1	48.1	39.7	5.61	4.35	5.71	22.8	33.8	22.4	40.4	23	12.7	37.3	60.1	61.5	27.2	1.7	9.54	28.1	51.6	18.7
CD44	92.5	94.2	96.1	93.1	98.8	93.9	10.4	14.7	21.2	9.5	35.6	2.7	58.5	69.8	21	73.9	86.9	87.4	14.1	39.4	4.86	27.9	92.1	14.8
CD148	73.5	91.7	91	90.8	96.9	92.6	13.7	29.2	21.4	27.1	28.2	11.2	93.8	99.1	37.5	86.5	91.6	96.6	79.1	73.8	30.6	51.7	90.1	61.4

Endothelial and fibroblast cluster

	229 CD45+	243 CD45+	266 CD45+	267 CD45+	278 CD45+	284 CD45+	229 Endothel	243 Endothel	266 Endothel	267 Endothel	278 Endothel	284 Endothel	229 Fibroblas	243 Fibroblas	266 Fibroblas	267 Fibroblas	278 Fibroblas	284 Fibroblas	229 Lin-	243 Lin-	266 Lin-	267 Lin-	278 Lin-	284 Lin-
CDW93	30	6.18	13.3	14.2	24.6	27.4	73	74.4	96	70.1	64.9	53.2	50.9	75.6	15.6	36.4	47.6	52	30.4	11.1	9.17	17.3	26.5	20.7
CD39	27.7	31.3	26.1	35.8	29.9	19.5	98.1	83.9	100	93.9	98.4	98.5	80.1	86.8	17.5	65.6	97	89.4	0.443	1.15	0.588	0.563	1.43	4.27
CD141	22.1	15	19.8	36.7	20.9	29.6	96.8	100	97.5	99.5	100	99.5	71.5	86.8	14.7	90.3	95.1	96	2.92	43.2	3.67	48.9	42.7	15.6
CD130	7.58	4.33	6.92	15.4	12.3	3.78	47.7	96.2	74.1	72.5	85.2	68.4	64.1	51.6	22.2	52.9	80.8	63	33.3	23	20.9	45.6	28.6	29.7
CD34	3.2	2.52	1.49	4.99	0.747	5.85	99.8	99.1	100	100	100	100	97.9	96	35.1	74.7	96.7	90.1	3.82	8.24	4.58	63.2	0.833	20.2
CD146	21.4	11	12.1	20.7	16.1	19.8	100	98.7	97.3	100	98.6	99.5	96.9	99.2	64.2	98.9	100	98	34.9	25.3	31.2	66.4	36.9	62.5
CD107A	9.97	13.9	6.3	15.7	9.93	12.9	45.1	73.2	57.1	56	54.9	64.2	61.8	32.5	7.44	39.8	53	50.5	10.7	9.31	3.62	16.4	5.43	32
CD165	11.6	2.32	0.524	5.53	1.46	4.45	66.7	78.9	41.5	90.5	75.4	73	93	23.1	2.9	46.7	93.2	79.9	0.486	0.199	1.16	1.58	0.664	2.66
CD317	30.7	40.1	52.8	55.1	45.6	24.9	99.2	100	100	78.2	87.8	80	91.3	100	50	62.4	99.2	87.4	8.92	29.8	1	29.7	77.9	63.6

Immune, endothelial and fibroblast cluster

	229 CD45+	243 CD45+	266 CD45+	267 CD45+	278 CD45+	284 CD45+	229 Endothel	243 Endothel	266 Endothel	267 Endothel	278 Endothel	284 Endothel	229 Fibroblas	243 Fibroblas	266 Fibroblas	267 Fibroblas	278 Fibroblas	284 Fibroblas	229 Lin-	243 Lin-	266 Lin-	267 Lin-	278 Lin-	284 Lin-
CD74	43	20	26.1	36.6	18.8	0.582	11.6	8.82	74.1	11.3	53.2	0.405	31.6	13.7	11	9.41	38.4	1	2.34	0.861	3.26	3.03	3.49	0.358
CD85J	48.7	21.6	40.4	58.5	20	31.6	46.3	27.2	62.1	55.4	32.9	13.8	39.5	1.95	19.8	45.4	10.7	0.741	22.9	0.877	26.4	39.3	1.74	0.923
CD84	76.5	71.3	67.7	82.2	86.5	78.4	89.7	79.1	81.2	89.5	82.9	77.7	89.4	92.8	18.4	93.5	93	95.7	26.8	3.95	21	52.1	73	49.1
CD120a	38.9	33.1	14.2	65	31.1	12.8	11.6	41.3	56.5	43.5	43.3	26.2	41.3	48.6	12.8	53.8	50	24.3	3.78	5.01	7.87	26.6	18.1	14.1
CD143	33.1	29.7	15.5	65.7	25.5	14.5	70.6	100	97.1	100	69.1	75.3	45.1	97	16.1	80.7	41.7	24.8	2.22	2.74	18.1	34.7	17.1	11.5
CD31	65.3	77.7	73.3	76.5	55.3	60.3	100	96.8	100	100	100	99.7	60.8	68.7	61.5	69.1	48.6	68.3	11	4.41	27.6	30.7	8.1	20.6
CD102	38.4	33.4	68.4	43.8	53.7	54.1	99.5	99.4	100	99.7	97.8	99.2	48.5	51.1	20.2	17.4	76	41.7	6.38	1.95	6.76	10.1	47.5	1.96
CD298	81.7	91.6	95.8	97.6	98	0.181	100	100	100	100	100	99.6	97.8	51.7	51.3	92.5	97.6	1	53.7	23.5	18.4	37.1	52.8	0.108

Endothelial, fibroblast and cancer cluster

	229 CD45+	243 CD45+	266 CD45+	267 CD45+	278 CD45+	284 CD45+	229 Endothel	243 Endothel	266 Endothel	267 Endothel	278 Endothel	284 Endothel	229 Fibroblas	243 Fibroblas	266 Fibroblas	267 Fibroblas	278 Fibroblas	284 Fibroblas	229 Lin-	243 Lin-	266 Lin-	267 Lin-	278 Lin-	284 Lin-
CD66e	61.9	36	47.1	70.2	59.8	48	99.4	86.7	95.7	96.1	98.6	98	99.5	93.7	79.1	95.2	98.5	99.1	76.4	62.1	54.3	94.3	89.1	86
CD175s	52.9	22.6	40.2	56.1	32.6	35.1	99	84.2	100	93.9	100	97.4	97.9	78.9	89.7	96.7	98.9	99.4	57.5	30.3	66.1	62.8	80.6	75
CD105	38.8	36	60.4	63.6	35.7	47.3	99.7	100	100	100	98.5	100	99	100	83	89.6	100	97.5	61.9	11.5	63.7	64.6	62.8	57
CD223	42.3	24.5	54.1	59.2	56.7	31.7	100	100	100	100	100	99.6	100	95	98.4	97.4	100	99.3	83.2	6.64	87.1	82	77.4	51.7
CD283	27.5	13	43.2	37.1	30.1	3.69	76.5	75	94.6	81.5	73.9	66.2	77.2	71	55.3	75.5	65.6	60.9	41.8	6.88	53.7	43.9	51.5	10.5
CD51,2f,CI	23.1	4.9	7.42	20.2	4.97	7.9	90.4	85.7	81.5	92.9	86.1	93.4	95.5	98.6	25	96	100	99.3	90.5	56.9	16.8	86.1	52.4	85.3
CD61	13.4	4.26	1.93	12.2	3.93	12.9	77.1	100	92.9	100	84.1	88.8	96.6	93.8	10.5	100	100	99.5	81.8	53.3	1	92.6	37.4	81.9
CD200	17.6	3.75	5.41	6.25	13.7	6.34	94.2	82.9	100	96.4	100	97.7	98.3	75.7	53.5	71.3	96.8	94.5	99.4	72.7	77	8.37	99.5	59
CD9	74	22.3	21.9	52.7	85.4	37.6	100	95.1	98.2	97.2	100	98.3	99.6	92.1	93.2	99.6	99.5	98.5	96.6	81.3	97.4	96.1	100	92.2
CD49A	32.6	10.1	23.4	27.5	42.4	35.3	98.8	100	100	100	97.3	100	100	98.2	100	99.4	100	100	99.4	57.9	98.5	96.2	99.7	91.5
CD49B	19.5	15.7	20.2	12.6	27.5	14.4	100	99	100	99.5	100	99.5	97.7	94.4	100	92.6	93.8	99.1	99.4	84.8	95.3	98.5	99.7	91.8
CD49C	31	11.9	15.8	21.2	62.1	50.4	99.5	97.1	100	96.1	97.8	99.6	98.5	98.1	100	100	100	99.1	98.4	95.7	97.4	95.3	100	91.2
CD49F	48.9	45.9	45.4	75.1	65.6	53.9	100	100	100	100	100	99.6	95.9	100	100	100	98.8	99.2	98.6	85.9	98	97.2	99.7	88.9
CD63	63.8	40.2	52.6	71.9	68.9	71.3	99.5	98.7	100	100	100	100	99.6	98.2	92.2	100	100	100	99.5	96.7	84.5	97.6	99.9	94.8
CD304	14.6	2.94	23.4	30.7	10.7	14.4	94.3	72.7	86.8	94.9	95.6	94.5	100	87.2	78.3	99.6	100	100	99.1	44.6	72.1	88.6	97	84
